# Hsp72 (HSPA1A) Prevents Human Islet Amyloid Polypeptide Aggregation and Toxicity: A New Approach for Type 2 Diabetes Treatment

**DOI:** 10.1371/journal.pone.0149409

**Published:** 2016-03-09

**Authors:** Paola C. Rosas, Ganachari M. Nagaraja, Punit Kaur, Alexander Panossian, Georg Wickman, L. Rene Garcia, Fahd A. Al-Khamis, Alexzander A. A. Asea

**Affiliations:** 1 Division of Investigative Pathology, Scott & White Hospital and the Texas A&M Health Science Center, College of Medicine, Temple, Texas, United States of America; 2 Department of Microbiology, Biochemistry & Immunology, Morehouse School of Medicine, Atlanta, Georgia, United States of America; 3 University of Texas MD Anderson Cancer Center, Houston, Texas, United States of America; 4 Department of Research, Swedish Herbal Institute, Åskloster, Sweden; 5 Department of Biology and Howard Hughes Medical Institute, Texas A&M University, College Station, Texas, United States of America; 6 Department for Neuroscience Research, Institutes for Research & Medical Consultancies (IRMC) and Deanship for Scientific Research, University of Dammam, Dammam, Saudi Arabia; Texas A&M University Health Science Center College of Medicine & Baylor Scott and White Health, UNITED STATES

## Abstract

Type 2 diabetes is a growing public health concern and accounts for approximately 90% of all the cases of diabetes. Besides insulin resistance, type 2 diabetes is characterized by a deficit in β-cell mass as a result of misfolded human islet amyloid polypeptide (h-IAPP) which forms toxic aggregates that destroy pancreatic β-cells. Heat shock proteins (HSP) play an important role in combating the unwanted self-association of unfolded proteins. We hypothesized that Hsp72 (HSPA1A) prevents h-IAPP aggregation and toxicity. In this study, we demonstrated that thermal stress significantly up-regulates the intracellular expression of Hsp72, and prevents h-IAPP toxicity against pancreatic β-cells. Moreover, Hsp72 (HSPA1A) overexpression in pancreatic β-cells ameliorates h-IAPP toxicity. To test the hypothesis that Hsp72 (HSPA1A) prevents aggregation and fibril formation, we established a novel *C*. *elegans* model that expresses the highly amyloidogenic human pro-IAPP (h-proIAPP) that is implicated in amyloid formation and β-cell toxicity. We demonstrated that h-proIAPP expression in body-wall muscles, pharynx and neurons adversely affects *C*. *elegans* development. In addition, we demonstrated that h-proIAPP forms insoluble aggregates and that the co-expression of h-Hsp72 in our h-proIAPP *C*. *elegans* model, increases h-proIAPP solubility. Furthermore, treatment of transgenic h-proIAPP *C*. *elegans* with ADAPT-232, known to induce the expression and release of Hsp72 (HSPA1A), significantly improved the growth retardation phenotype of transgenic worms. Taken together, this study identifies Hsp72 (HSPA1A) as a potential treatment to prevent β-cell mass decline in type 2 diabetic patients and establishes for the first time a novel *in vivo* model that can be used to select compounds that attenuate h-proIAPP aggregation and toxicity.

## Introduction

Recently, the number of people with diabetes worldwide reached 382 million and by 2035 this number will increase to 592 million (International Diabetes Federation); 90% of whom have type 2 diabetes. An important characteristic of type 2 diabetes is a deficit in β-cell mass as a result of misfolded human islet amyloid polypeptide hormone (h-IAPP, also called amylin), which forms toxic aggregates that destroy pancreatic β-cells [1]. β-cell apoptosis begins early in the disease process and at diagnosis 40–60% of β-cell volume may already have been lost [2]. h-IAPP is a 37-amino acid hormone co-secreted with insulin by pancreatic β-cells, and is initially synthesized as a 89-amino acid pre-prohormone containing a 22 amino-acid signal peptide and two short flanking peptides that are later cleaved [3]. The signal peptide is cleaved in the endoplasmic reticulum (ER), where pre-proIAPP is converted into proIAPP and then converted into IAPP in secretory granules [3]. ProIAPP by itself is highly amyloidogenic and its aggregation could be the starter event of amyloid formation [4, 5]. *In vitro* studies demonstrated that proIAPP aggregates are toxic to pancreatic beta cells [6]. Immune electron microscopic studies with antibodies specific to proIAPP, evidenced the presence of proIAPP together with IAPP in intracellular amyloid [4]. Human, feline and non-human primate forms of IAPP are the only forms with the capacity to oligomerize and form aggregates.

Insulin resistance and hyperglycemia characteristics of type 2 diabetes not only induce insulin production but also increase IAPP production, which demands higher concentrations of molecular chaperones to help with folding activities. This higher demand for chaperones, within β-cells already exhausted due to hyperglycemic stress, may result in β-cell overload ultimately leading to the accumulation of misfolded IAPP aggregates and toxicity. Moreover, availability of chaperones are decreased in states such as cell stress and aging [7]. Therefore, increasing the levels of chaperones in order to protect pancreatic β-cells against cell damage provides an attractive therapeutic target for the treatment of type 2 diabetes. Especially important is Hsp72 (HSPA1A), the stress-inducible form of the HSP70 super-family, that acts early in the life of proteins binding to short hydrophobic segments preventing misfolding and aggregation. Hsp72 (HSPA1A) also has refolding activities and promotes the degradation of misfolded proteins and aggregates [8]. Among the substances that induce the expression of Hsp72 (HSPA1A), is a group of plant extracts called adaptogens characterized by their property to promote a non-specific adaptive response to resist stressful conditions [9]. The most extensively studied adaptogens are: *Eleutherococcus senticocus*, *Schisandra chinensis* and *Rhodiola rosea*, which are the active ingredients of ADAPT-232 [10]. Here we present evidence that ADAPT-232 has the potential for translation to a novel therapy to prevent IAPP toxic aggregation.

In this study, *Caenorhabditis elegans* (*C*. *elegans*) was chosen as a model system to characterize the aggregation of h-proIAPP and to establish the role of molecular chaperones in preventing or ameliorating h-proIAPP aggregation over the traditional rat models for diabetes for several reasons: Onset of diabetes in the traditional rat model takes weeks as compared to days in *C*. *elegans*. The *C*. *elegans* model system allows researchers to more easily study the effect of diabetes over the entire life span of the animal, since it has a relatively short life cycle of approximately 3 days. The size of *C*. *elegans* (1 mm long) and transparency allows the expression and easy visualization of fluorescently tagged proteins. *C*. *elegans* has also previously been used as an *in vivo* model system to study diseases related with protein aggregation such as neurodegenerative diseases [11]. Finally, the *C*. *elegans* model system is less expensive to purchase and maintain than the traditional rat model and the large number of eggs laid by *C*. *elegans* hermaphrodites allows cultivation of a large number of animals in a short period of time. Also, the worms are easy to maintain in the laboratory using agar plates with *E*. *coli* as a food source. Here, we demonstrated that h-proIAPP expression in body-wall muscles, pharynx and neurons results in a growth retardation phenotype that was significantly improved by treatment with ADAPT-232. Moreover, h-proIAPP forms insoluble aggregates inside the worm and the co-expression of human Hsp72 (h-Hsp72) increases proIAPP solubility.

## Materials and Methods

### Institutional animal care & use committee (IACUC) and animal research ethics statement

Work with the invertebrate nematode model organism, *C*. *elegans*, does not require Institutional Animal Care and Use Committee (IACUC) approval, as stated by the Public Health Service Policy on Humane Care and Use of Laboratory Animals (PHS).

### Cells and culture conditions

Beta-TC-6 cells (ATCC, Manassas, VA) were maintained in monolayer cultures in HEPES-buffered Dulbecco’s modified Eagle’s medium (Life Technologies, Grand Island, NY) supplemented with 15% heat-inactivated fetal bovine serum (Life Technologies), 100 IU/ml penicillin and 100 μg/ml streptomycin (Life Technologies). Cells were maintained at 37°C humidified atmosphere with 5% CO_2_. h-IAPP (Bachem, King of Prussia, PA) was dissolved in water and immediately added it to the culture medium of β-cells at various concentrations.

### Cell viability assays

Cell viability was measured in cells plated on 96-well tissue culture plates by MTS assay (Promega Corp., Madison, WI). MTS [3-(4,5-dimethylthiazol-2-yl)-5-(3-carboxymethoxyphenyl)-2-(4-sulfophenyl)-2H-tetrazolium, inner salt] was utilized according to the manufacturer’s instructions.

### Western blot analysis

Total cell extracts (50μg) from Beta-TC-6 cells were isolated according to standard protocol (Cell Signaling Technology, Danvers, MA); fractionated by electrophoresis on 12% SDS polyacrylamide gels; electroblotted to PVDF membrane (GE healthcare, Piscataway, NJ) and probed with anti-Hsp72 (Enzo Life Sciences, Farmingdale, NY), and anti-Hsp25 (Enzo Life Sciences) antibodies. To detect IAPP expression, membranes were probed with a polyclonal antibody raised against amino acids 40–89 of IAPP precursor of human origin (Santa Cruz Biotechnology, Dallas, TX) that cross reacts to a lesser extent with mouse IAPP. Protein loading control used was β-actin (Abcam, Cambridge, MA). Appropriate horseradish peroxidase-conjugated secondary antibodies (Jackson ImmunoResearch, West Grove, PA) were used in the study. Protein bands were visualized by chemiluminescence (Thermo Scientific, Rockford, IL), films were developed and image processor Quantity One (Version 4.6) was used to scan and to compare expression levels.

### Transfection procedures

Beta-TC-6 cells were plated on 96-well tissue culture plates and allowed to attach overnight. When 70–80% confluency was reached, cells were transfected with h-proIAPP cDNA clone (vector pCMV6-XL5, Origene, Rockville, MD) or m-proIAPP cDNA clone (vector pCMV6-Kan/NEO, Origene) or h-Hsp72 cDNA clone (vector pEGFP, Addgene, Cambridge, MA) or both h-proIAPP and h-Hsp72 vectors. Transfection complexes were prepared with Opti-MEM (Life Technologies), and Lipofectamine LTX and PLUS reagent (Life Technologies), according to the manufacturer’s instructions. Medium was changed after 4–6 hours. After 48 hours of incubation we used MTS assay to evaluate cell viability.

### *C*. *elegans* strains and culture techniques

The following strains of *C*. *elegans* used in this study including, *pha-1(e2123)* [12]; *him-5(e1490)* [13]; and *lite-1(ce314)* [14] were obtained from Prof. Renee Garcia (Texas A&M University). Animals were maintained on NGM plates seeded with *Escherichia coli* strain OP50 at 20°C [15]. Further details on maintenance and culture techniques can be found in the Supporting Information section.

### Constructs

Human and mouse preproIAPP cDNA clones (Origene, Rockville, MD) were subcloned into BamH1 sites of expression vector pSX95.77YFP using the In-Fusion^TM^ PCR cloning system (Clontech, Mountain View, Ca). For h-proIAPP cloning we use h-proIAPP forward (5′-CGACTCTAGAGGATCCATGGGCATCCTGAAGCTGCAAG-3′) and reverse (5′-CCAATCCCGGGGATCCAAGGGGCAAGTAATTCAGTGG-3′) primers. For mouse-proIAPP (m-proIAPP) cloning we use m-proIAPP forward (5′-CGACTCTAGAGGATCCATGATGTGCATCTCCAAACTGCCAGC-3′) and reverse (5′-CCAATCCCGGGGATCCAACGAGTAAGAAATCCAAGG-3′) primers. The resulting constructs were digested with Sal I, blunt-ended and then ligated with the Gateway Vector Conversion Reading Frame Cassette C.1 (Life Technologies, Grand Island, NY) to generate Gateway destination vectors pNG1 and pNG2 that contained the human and mouse preproIAPP clones, respectively. Entry clones pLR22, pLR25 and pLR35 contained the *C*. *elegans* promoter regions of *lev-11* (body-wall muscles), *tnt-4* (pharynx) and *aex-3* (pan-neuronal), respectively. Promoter sequences in pLR22, pLR25 and pLR35 were recombined into pNG1and pNG2 using LR recombination reaction, to generate *C*. *elegans* transgenic tissue-specific plasmids. Three human IAPP plasmids: pPR3 *(lev-11* promoter), pPR4 *(tnt-4* promoter) and pPR5 (*aex-3* promoter); and three mouse IAPP plasmids: pPR8 *(lev-11* promoter), pPR9 (*tnt-4* promoter) and pPR10 (*aex-3* promoter) were generated. To generate a tissue-specific expression of YFP, construct pSX95.77YFP was digested with Sal I, blunt-ended and ligated with Gateway C.1 cassette to generate pNG3. The promoter sequence in pLR22 (*lev-11* promoter, body-wall muscles) was recombined into pNG3, using LR recombination reaction to generate plasmid pPR18. To generate tissue-specific expression of the h-Hsp72 plasmid; the h-Hsp72 insert was amplified using the pOTB7 vector (Life Technologies) that contained the Hsp72 clone. The cDNA fragments were subcloned into BamH1 sites of expression vector pTG24 by In-Fusion^TM^ PCR cloning using h-Hsp72 forward (5′-CGACTCTAGAGGATCCATGGCCAAAGCCGCGGCGATCGG-3′) and reverse (5′-CCAATCCCGGGGATCCATCCACCTCCTCAATGGTGGG-3′) primers. The resulting constructs were digested with Xbal, blunt-ended and ligated with Gateway C.1 cassette to generate pNG4 that contained the h-Hsp72 sequence. Entry clone pLR22 was recombined using LR recombination reaction into pNG4 to generate tissue specific plasmid pPR21 *(lev-11* promoter). For a complete list of primers used in this study see S1 Table.

### Transgenics

Plasmids containing the construct of interest were injected into 1-day-old adult *pha-1(e2123)*; *him-5(e1490)*; *lite-1(ce314)* hermaphrodites following standard procedures [16]. 50 ng/μl of the pha-1 rescuing plasmid, pBX1, was used as a transgenic marker, as previously described [17]. pUC18 was used as carrier DNA to bring each injection mixture to a final concentration of 200 ng/μl.

### Growth retardation phenotype

Ten to fifteen gravid 1- to 3-day-old adult hermaphrodites were placed on NGM agar plates or NGM ADAPT-supplemented plates with OP50 as a food source and treated with one drop of bleaching solution in order to induce the release of the eggs. Larvae were synchronized and seventy-two hours after treatment, larvae stage or adult stage was determined.

### Image acquisition

One-day-old adult animals were paralyzed using sodium azide, mounted on 2% agarose pads and covered with a cover slip. Images of fluorescent animals were taken using an Olympus Fluoview 300 confocal microscope with a 60X apochromat water immersion objective. The YFP-labeled structures were excited at 488 nm and the fluorescence was collected through a 560–600 nm band-pass filter. All images were taken at 10% power intensity; PMT, gain and offset adjusting were held constant. Each confocal slice consisted of 512 (X) x 512 (Y) pixels. We scanned completely through the animals at 0.5μm Z-axis increments. Mean fluorescence intensity was measured using a sum intensity projection in ImageJ on all the planes of the acquired stacks of the different structures. Maximum intensity projections were created using ImageJ on all the planes of the acquired stacks to output images for visualization. Differential interference contrast (DIC) images using 60X magnification were taken to visualize structures.

### Fluorescence recovery after photobleaching (FRAP) analysis

FRAP analysis was performed as previously described [18, 19] and described in detail in the Supporting Information section. Images were obtained using the Olympus Fluoview 300 confocal microscope and analyzed using ImageJ.

### Statistical analysis

Data were evaluated for statistical significance using Student *t*-test for pair wise comparison of two groups and ANOVA post-hoc Tukey test for comparison of 3 groups. Data are expressed as the mean ± SD, unless otherwise specified.

## Results

### Heat shock treatment reduces exogenous h-IAPP toxicity against pancreatic β-cells

**Fig 1:**
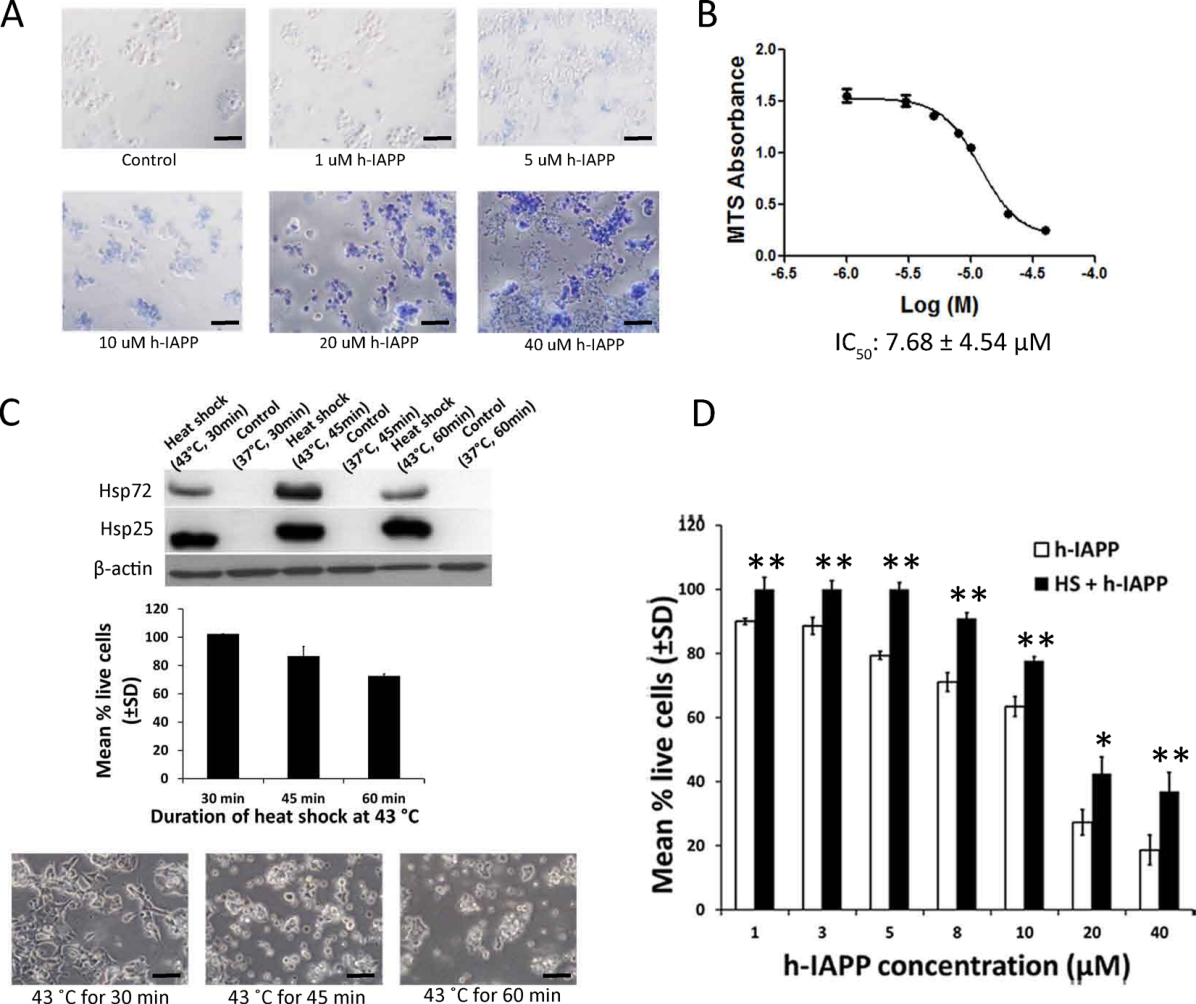
Heat shock treatment protects pancreatic β-cells against h-IAPP toxicity. **A.** Beta-TC-6 cells were exposed to various concentrations of exogenously added h-IAPP for a 24 hours period. Cell viability was determined by trypan blue exclusion assay. Live cells exclude trypan blue, while dead cells take up trypan blue and appear blue when observed under a white light microscope. Data is a representative experiment from at least three independently performed experiments with similar results. Scale bars represent 100 μm. **B.** Beta-TC-6 cells were exposed to various concentrations of h-IAPP. Twenty-four hours later, cell viability was measured using MTS cell proliferation assay. IC_50_ was calculated as the concentration of h-IAPP required to obtain 50% of its maximum toxic effect (± SD). **C.** Beta-TC-6 cells were exposed at 43°C for 30, 45 and 60 minutes and the expression of Hsp72 and Hsp25 were measured by Western blot 24 hours after heat exposure (top panel). Beta-TC-6 cells were exposed to heat shock at 43°C for 30, 45 and 60 minutes and cell viability was determined by MTS assay 24 h after heat exposure (middle panel; filled bars). Phase contrast microscopy of Beta-TC-6 cells exposed to heat shock at 43°C for 30, 45 and 60 minutes 24 h after heat exposure (bottom panel). Data is a representative experiment from at least three independently performed experiments with similar results. Scale bars represent 50μm. **D.** Heat treatment at 43°C for 30 minutes protects Beta-TC-6 cells against exogenous h-IAPP toxicity. Beta-TC-6 cells were heat shocked at 43°C for 30 min (filled bars) or maintained at normal temperature 37°C for 30 min (open bars). On day 2, cells were treated with various concentrations of h-IAPP. After 24 h of incubation, cell viability was assessed by MTS assay. Data represents the sum of three of three independently performed experiments. *p<0.05; **p<0.01 versus respective controls, n = 3.

Mouse insulinoma Beta-TC-6 cells (ATCC) were exposed to various concentrations of exogenous h-IAPP. Similar to the report by Lorenzo et al. [20], toxicity was initially detected at a concentration of 5 μM h-IAPP (determined by trypan blue exclusion assay) and complete cell death occurred at 40 μM (Fig 1A). In order to quantitatively measure h-IAPP toxicity against Beta-TC-6 cells, we calculated h-IAPP IC_50_ using MTS cell proliferation assay (Promega). The IC_50_ for h-IAPP was calculated at 7.68 ± 4.54 μM (Fig 1B). To select the optimum heat shock treatment to be used in the prevention of h-IAPP toxicity, we evaluated the expression levels of inducible chaperones due to thermal stress on Beta-TC-6 cells. We found that the levels of Hsp72 (HspA1A) and Hsp25 (HspB1) are absent under basal conditions and their expression was up-regulated after thermal stress, with the highest levels found after 45 min of heat shock at 43°C (Fig 1C; top panel). We also found that heat shock at 43°C affects Beta-TC-6 cells viability, as determined by MTS assay 24 hours post heat shock treatment (Fig 1C; middle and bottom panels). Cell viability was not affected after 30 min of heat shock; thus, this treatment was selected to evaluate the role of Hsp72 (HSPA1A) in preventing h-IAPP toxicity. Having demonstrated that heat stress increased the levels of Hsp72 (HSPA1A), we then established whether this induction protects β-cells against exogenously added h-IAPP. For this purpose, Beta-TC-6 cells were plated and 24 hours later were heat shocked at 43°C for 30 min and then returned to the incubator for an additional 24 hours to allow for Hsp72 expression. Another set of cells were kept at 37°C for 30 min to serve as a control. On day 3, various concentrations of h-IAPP solution were applied to the cell culture medium. After 24 h of incubation, cell viability was assessed by MTS assay. We demonstrated that heat shock significantly reduced the toxic effect of h-IAPP (Fig 1D). In a separate experiment, we demonstrated that there is a significant variation in fluorescence intensity of cells expressing DsRed-h-IAPP (S1 Fig). We further demonstrated that cells that expressing the highest red fluorescence intensity were round and detached suggesting that they were dead due to h-IAPP toxicity (S1 Fig).

### Hsp72 ameliorates toxicity induced by endogenous expression of h-IAPP in pancreatic β-cells

**Fig 2:**
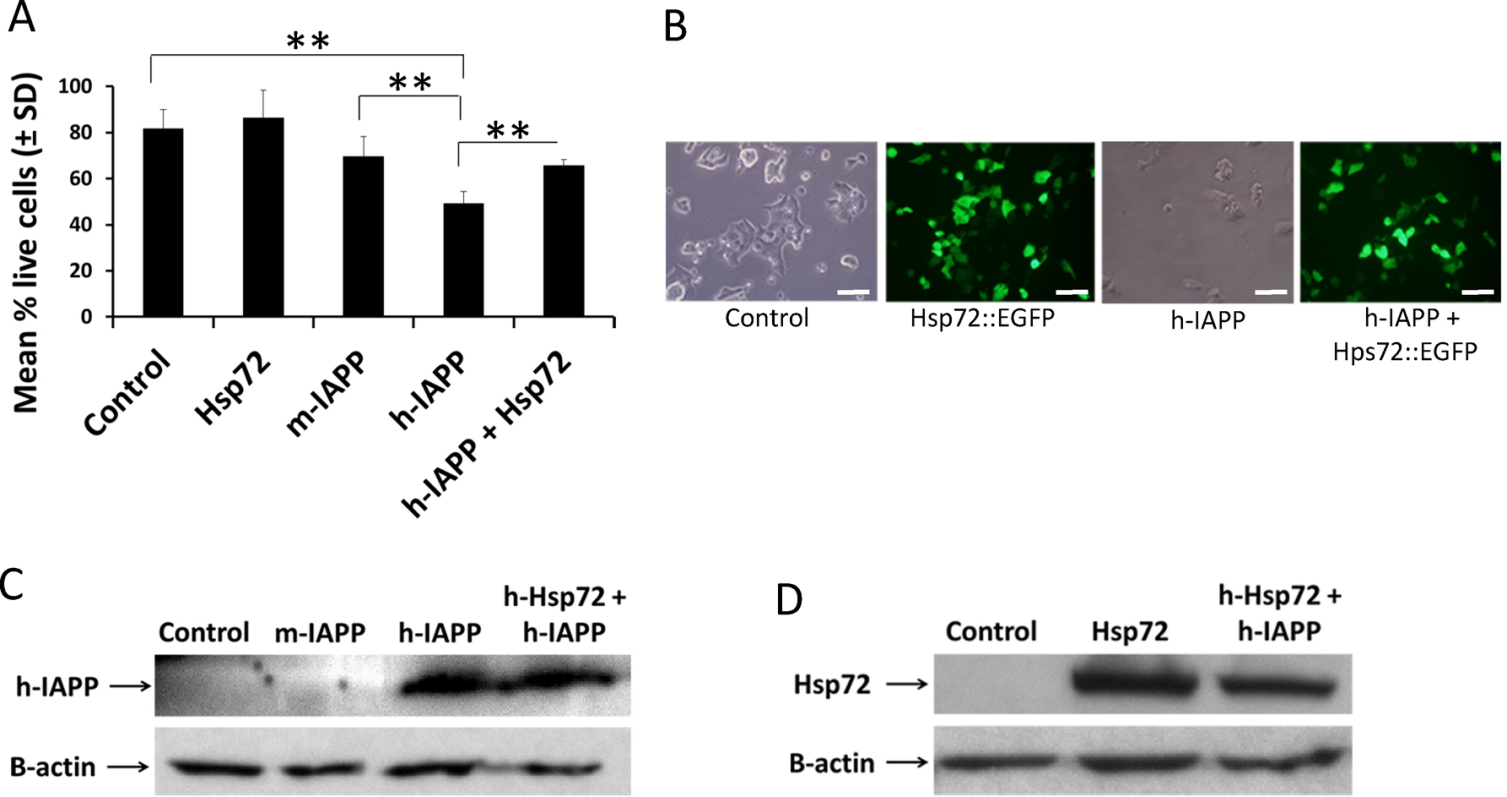
Hsp72 reduces endogenous h-IAPP toxicity in pancreatic β-cells. **A.** Beta-TC-6 cells were transfected and cell viability was assessed by MTS assay. Data represent percentage of live cells ± SD (filled bars) when transfected with vector pCMV6-XL5 (control) or human Hsp72::EGFP cDNA clone or m-proIAPP cDNA clone or h-proIAPP cDNA clone or both h-proIAPP and human Hsp72::EGFP vectors. Data represents the sum of three independently performed experiments. **p<0.01 versus respective controls, n = 3. **B.** At the end of the transfection period, phase contrast and fluorescence images were obtained. Data is a representative experiment from at least three independently performed experiments with similar results. Scale bars represent 50 μm. **C.** Beta-TC-6 cells were transfected with empty vector pCMV6-XL5 (control), or m-proIAPP cDNA clone or h-proIAPP cDNA clone or with both h-proIAPP and human Hsp72::EGFP vectors. After transfection, samples were collected and examined for the expression of h-IAPP by Western blot analysis. Data is a representative experiment from at least three independently performed experiments with similar results. **D.** Beta-TC-6 cells were transfected with empty vector pCMV6-XL5 (control), or human Hsp72::EGFP cDNA clone, or with both h-proIAPP and human Hsp72::EGFP vectors. After transfection, samples were collected and examined for the expression of Hsp72 by Western blot analysis. Data is a representative experiment from at least three independently performed experiments with similar results.

To establish the effect of endogenously expressed h-IAPP in Beta-TC-6 cells, we transiently transfected these cells with plasmids expressing human proIAPP (vector pCMV6-XL5; Origene) and mouse proIAPP (vector pCMV6-Kan/NEO, Origene), empty vector (pCMV6-XL5; Origene) served as a transfection control. Transfection complexes were prepared with Opti-MEM (Gibco), and Lipofectamine LTX with Plus reagent (Life Technologies), according to the manufacturer’s instructions. Medium was changed after 4–6 hours and cells were incubated for 48 hours. At the end of the transfection period, cell viability was assessed by MTS assay. We demonstrated that h-IAPP over-expression significantly induced Beta-TC-6 cell death as compared to controls, p<0.01 (Fig 2A and 2B); while over–expression of m-IAPP was less toxic (Fig 2A). To evaluate the role of Hsp72 in h-IAPP toxicity, we co-transfected Beta-TC-6 cells with h-proIAPP and Hsp72::EGFP plasmids; medium was changed after 4–6 hours and cells were incubated for 48 hours. At the end of the transfection period, cell viability was assessed by the MTS assay. This experiment revealed that h-Hsp72 over-expression significantly prevented h-IAPP-induced toxicity, as compared to cells transfected with h-IAPP alone, p<0.01 (Fig 2A and 2B). Western blot analysis did not detect any significant differences in the expression levels of h-IAPP or Hsp72 when transfected alone or in combination (Fig 2C and 2D). Meanwhile, the antibody used to detect IAPP was effective at detecting h-IAPP, but did not detect m-IAPP (Fig 2C).

### Constitutive expression of human but not mouse-proIAPP in *C*. *elegans* induces insoluble aggregates

**Fig 3:**
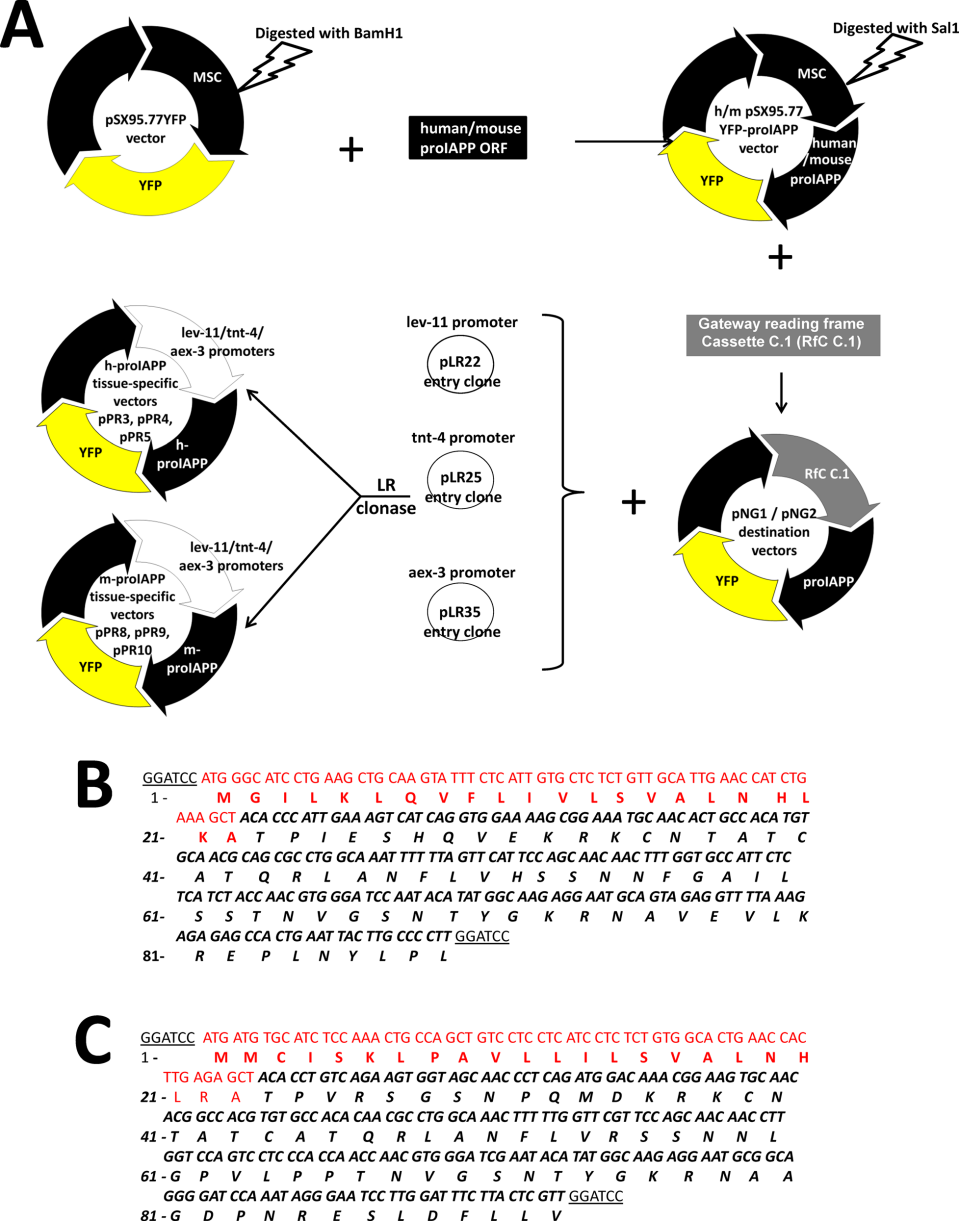
Construction and identification of human and mouse proIAPP *C*. *elegans* vectors. **A.** Schematic representation of human-proIAPP and mouse-proIAPP tissue-specific vector constructs. cDNA of human and mouse proIAPP with human and mouse signal peptide, respectively, were cloned into the BamH1 restriction site of the pSX95.77YFP *C*.*elegans* vector to generate human and mouse pSX95.77YFP-proIAPP transgenes. Resulting constructs were digested with Sal1, blunt-ended and ligated into Gateway Cassette C.1 upstream of human and mouse coding sequences to create vectors pNG1 and pNG2. Destination vectors pNG1 and pNG2 were LR recombined with pLR22, pLR25 and pLR35 entry clones to generate human proIAPP plasmids: pPR3, pPR4 and pPR5 and mouse proIAPP plasmids: pPR8, pPR9 and pPR10. **B.** h-proIAPP sequence of construct pSX95.77YFP-prohIAPP with human signal peptide. Preproh-IAPP (1–89) peptide amino acid sequence is shown in black italics. Signal peptide sequence is shown in red. **C.** m-proIAPP sequence present in construct pSX95.77YFP-promIAPP with mouse signal peptide. Preprom-IAPP peptide amino acid sequence is shown in black italics. Signal peptide sequence is shown in red. Restriction sites used in the generation of these plasmids are underlined. No stop codon was included after each proIAPP sequence.

To generate a proIAPP *C*. *elegans* model system, cDNA encoding h-proIAPP and m-proIAPP, containing the human and mouse signal peptide, respectively, were inserted in vector pSX95.77YFP, which contains yellow fluorescence protein gene positioned in front of the *C*. *elegans unc-54* 3’UTR (Fig 3A). The proIAPP genes were translationally fused to the 5’-end of the YFP coding sequence (Fig 3A). Positive clones were identified by colony PCR (S2 Fig) and sequenced (Fig 3B and 3C). Human and mouse pSX95.77YFP-proIAPP constructs were ligated into Gateway Cassette C.1 upstream of h-proIAPP and m-proIAPP coding sequences, to create vectors pNG1 and pNG2, respectively (Fig 3A). pNG1 and pNG2 were recombined with pLR22, pLR25 and pLR35 to create h-proIAPP plasmids: pPR3, pPR4 and pPR5 and m-proIAPP plasmids: pPR8, pPR9 and pPR10 (Fig 3A). Transgenic h-proIAPP *C*. *elegans* animals were generated by gonad microinjection of 20ng/μl of plasmids pPR3, pPR4 and pPR5. Transgenic m-proIAPP *C*. *elegans* animals were generated by gonad microinjection of 20ng/μl of plasmids pPR8, pPR9 and pPR10. *C*. *elegans* injected only with 50ng/μl of plasmid pBX1 was used as a negative control (pBX1 control).

**Fig 4:**
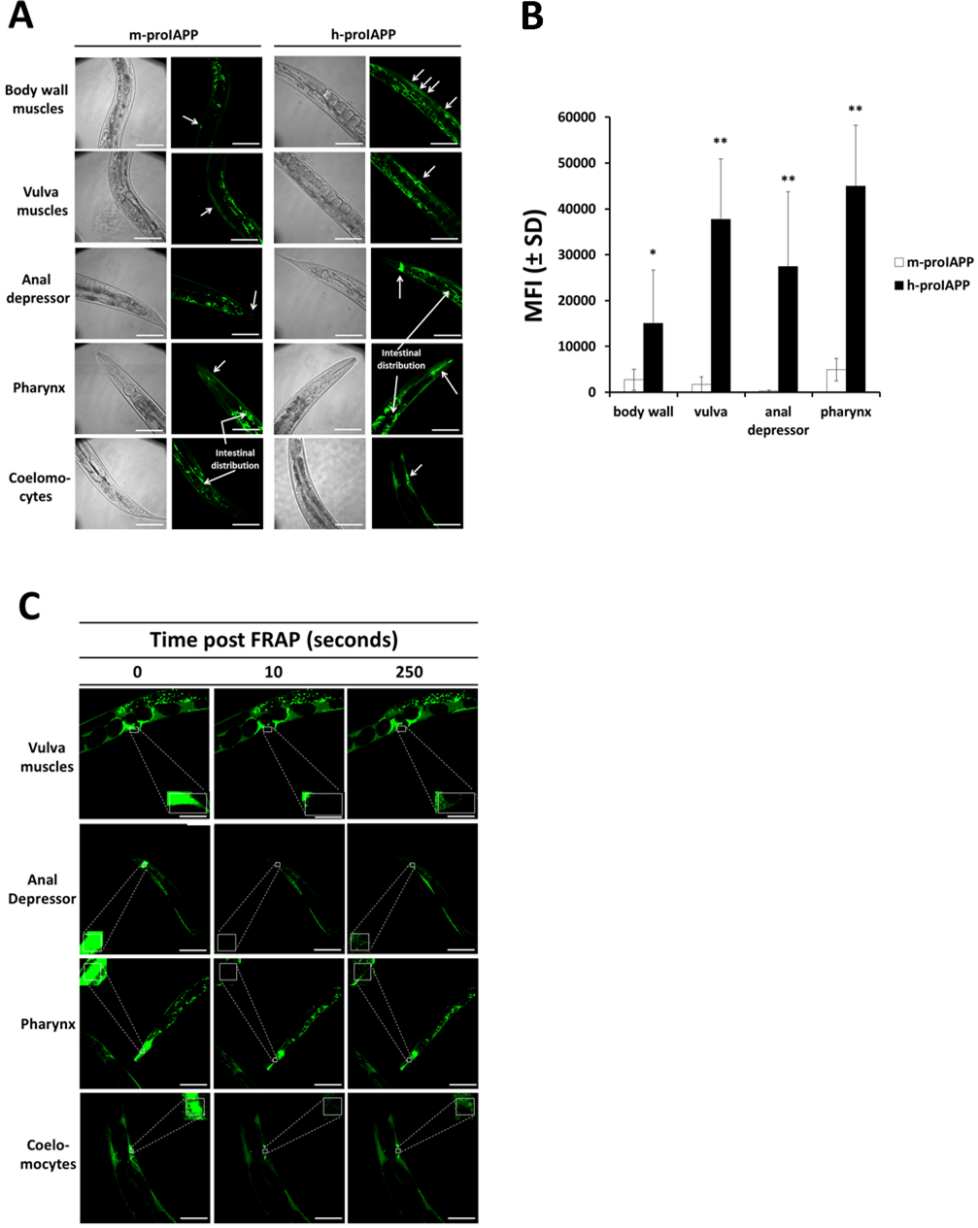
Expression of h-proIAPP in *C*. *elegans* results in protein insolubility and aggregation. Transgenic h-proIAPP *C*. *elegans* model was generated by gonad microinjection of 20 ng/μl of plasmids pPR3, pPR4 and pPR5. Transgenic m-proIAPP *C*. *elegans* model was generated by gonad microinjection of 20 ng/μl of plasmids pPR8, pPR9 and pPR10. **A.** h-proIAPP and m-proIAPP tagged with YFP were expressed in body wall muscles, vulva muscles and anal depressor muscles under *lev-11* promoter; in pharynx under *tnt-4* promoter, and in neurons under *aex-3* promoter. Neuronal expression of proIAPP secreted into the body cavity was observed in the coelomocytes of the h-proIAPP model and distributed in the intestinal region in both h-proIAPP and m-proIAPP *C*. *elegans* models. Images were created using maximum intensity projections on all the planes of the acquired stacks except coelomocytes where images were taken in a single plane for better visualization. Arrows indicate areas of fluorescence. DIC images were taken to visualize structures. Results are a representative experiment from at least three independently performed experiments with similar results. Scale bars represent 50 μm. **B.** Data represent the mean fluorescence intensity (MFI) ± SD measured using a sum intensity projection on all the planes of the acquired stacks of m-proIAPP (open bars) and h-proIAPP (filled bars) tagged with YFP expressed in body wall muscles, vulva muscles, anal depressor muscles and pharynx. *p<0.05; **p<0.01 versus m-proIAPP. **C.** Transgenic h-proIAPP *C*. *elegans* tagged with YFP were subjected to FRAP analysis (square). Data are from vulva muscles (row 1), anal depressor (row 2), pharynx (row 3) and coelomocytes (row 4) before FRAP (left panels), 10 seconds after FRAP (middle panels) 250 seconds after FRAP (right panels). Images were obtained using an inverted confocal microscope. Results are representative experiment from at least three independently performed experiments with similar results. Scale bars represent 50 μm.

We observed significantly higher fluorescence intensity in the animal body-wall muscles, vulva muscles and anal depressor muscles when h-proIAPP was expressed under the muscle-specific *lev-11* promoter (Fig 4A; right panels and Fig 4B), as compared to animals in which m-proIAPP was expressed under the same promoter (Fig 4A; left panels and Fig 4B). Similarly, h-proIAPP expression in pharynx muscles under the pharyngeal-specific *tnt-4* promoter, exhibited significantly higher fluorescence intensity (Fig 4A; right panels and Fig 4B) as compared to the m-proIAPP expression under the same promoter (Fig 4A; left panels and Fig 4B). Expression of proIAPP driven by the *aex-3* pan-neuronal promoter was secreted from neurons into the body cavity where it was taken up by coelomocytes, known to have a phagocytic function similar to macrophages of vertebrates. Coelomocytes found in the transgenic h-proIAPP *C*. *elegans* model were more intensely fluorescent (Fig 4A; right panels and S3 Fig) than coelomocytes in the m-proIAPP *C*. *elegans* model (Fig 4A; left panels). Differences in fluorescence intensity cannot be explained by differences in expression levels, since both h-proIAPP and m-proIAPP *C*. *elegans* models showed similar proIAPP mRNA levels determined by RT-PCR (S4 Fig). Expression of plasmids under the inducible hsp-16-2 promoter resulted in high expression of pro-IAPP in the pharynx, body wall muscles and intestinal walls after animals were exposed to heat stress at 33°C for 90 minutes (S5 Fig). Transgenic h-proIAPP *C*. *elegans* animals exhibited significantly more aggregates than transgenic m-proIAPP *C*. *elegans* animals, particularly in the pharynx (S5 Fig; right panels). To determine if the higher fluorescence intensity found in the transgenic h-proIAPP *C*. *elegans* model correspond to the formation of insoluble h-proIAPP aggregates, we used FRAP analysis. Fluorescent regions that do not recover after photobleaching were considered to contain immobile protein aggregates [18]. We demonstrated that h-proIAPP expression in *C*. *elegans* formed insoluble aggregates that do not diffuse to restore fluorescence of the photobleached region. This phenomenon was observed in vulva muscles, anal depressor muscles, pharynx and coelomocytes of animals expressing h-proIAPP (Fig 4C).

### Human-proIAPP-induced aggregates mediates developmental retardation in the *C*. *elegans*

**Fig 5:**
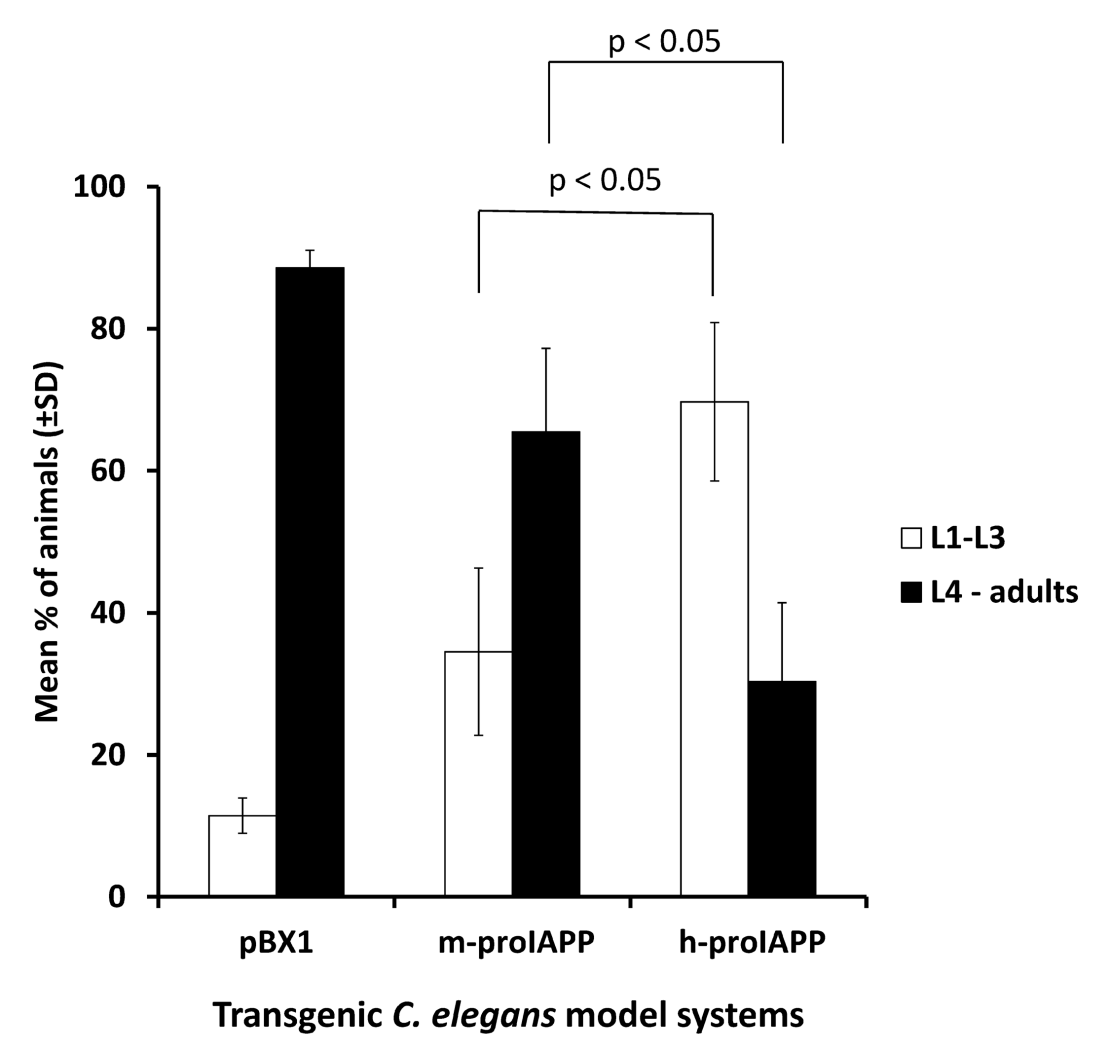
Expression of h-proIAPP in *C*. *elegans* correlates with growth retardation phenotype. Developmental phenotype was studied in transgenic h-proIAPP *C*. *elegans* model, transgenic m-proIAPP *C*. *elegans* model and pBX1 control animals by quantifying the number of animals in the larvae 1 (L1) to larvae 3 (L3) stages (open bars) versus larvae 4 (L4) and adult stages (filled bars) seventy-two hours after ten to fifteen gravid hermaphrodites were treated with bleach to release eggs. Data represent the percentage of animals found at larvae 1 to larvae 3 stages and the percentage of animals found at larvae 4 and adult stages ± S.D. p<0.05 versus respective control, n = 3.

We next examined the effect of h-proIAPP aggregates on *C*. *elegans* behavioral phenotypes. We initially measured life span of all three transgenic model systems (pBX1, m-proIAPP and h-proIAPP) and did not find any statistically differences (data not shown). We next measured the rate of development by synchronizing worms with hypochlorite treatment as described in detail in the Material and Methods section. Seventy hours after treatment, larvae stage was determined and compared with the pBX1 controls. We demonstrated significant developmental retardation in the transgenic h-proIAPP *C*. *elegans*, as compared to the m-proIAPP and pBX1 controls (Fig 5).

### Hsp72 expression improves the solubility of h-proIAPP in the *C*. *elegans* model

To demonstrate the effect of Hsp72 in protein aggregation, we constitutively co-expressed h-proIAPP tagged with YFP together with h-Hsp72 in *C*. *elegans* body-wall muscles (*lev-11* promoter). Transgenic h-Hsp72 and h-proIAPP *C*. *elegans* animals were generated by gonad co-injection of 20ng/μl of h-proIAPP tagged with YFP expressed in body-wall muscles (plasmid pPR3) and 30ng/μl of Hsp72 expressed in the same tissue (plasmid pPR21). *C*. *elegans* injected with 20ng/μl of plasmid pPR3 was used as a control for protein aggregation. To serve as a soluble control, a plasmid that carries only YFP expressed in body-wall muscles (plasmid pPR18) was created. YFP plasmid (pPR18, 20ng/μl) together with Hsp72 plasmid (pPR21, 30ng/μl) expressed in the same tissue were microinjected to generate a transgenic *C*. *elegans* model system that expresses both YFP and Hsp72 in body-wall muscles.

**Fig 6:**
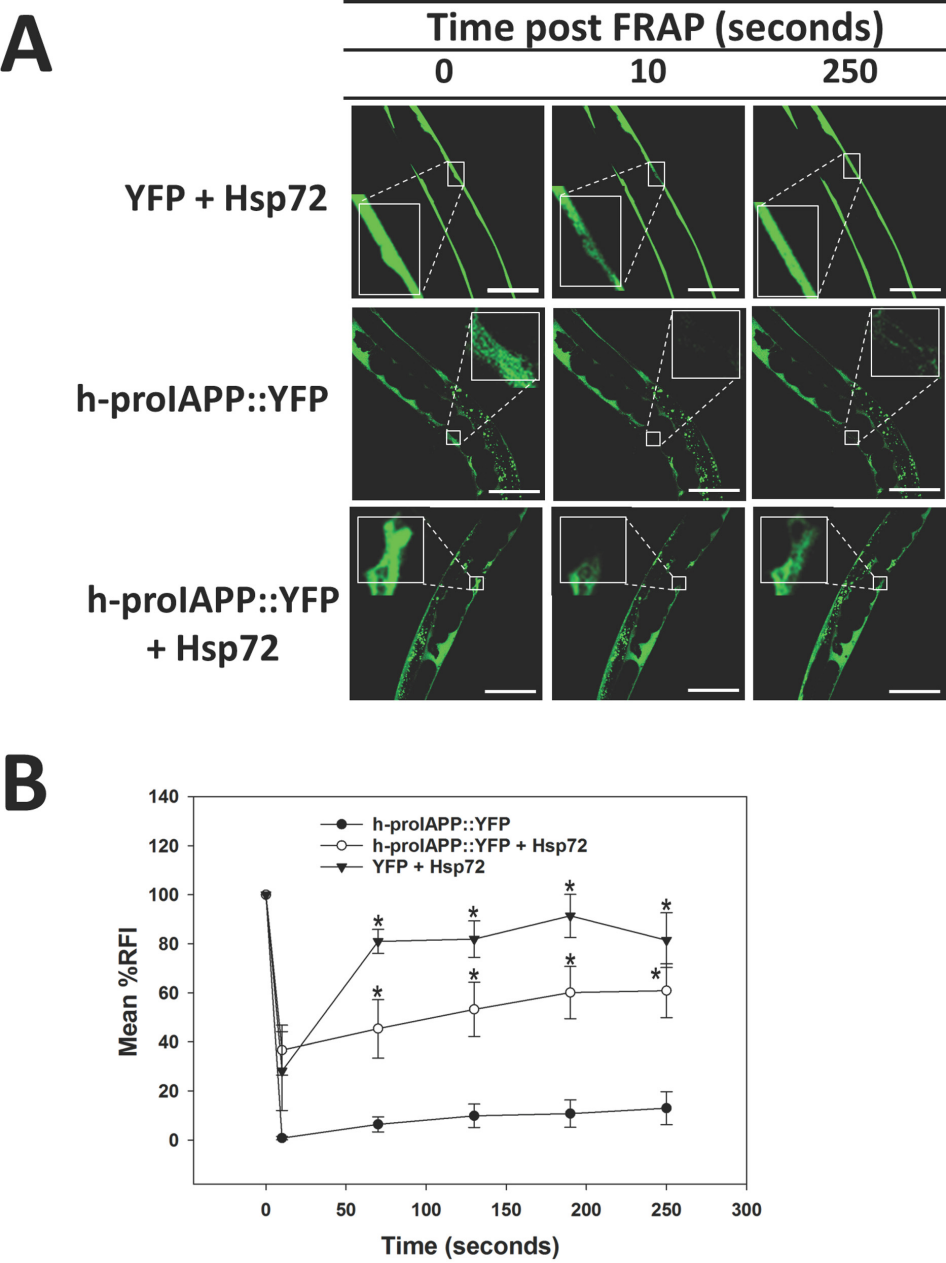
Hsp72 expression improves the solubility of h-proIAPP aggregates. **A.** Transgenic YFP + Hsp72 *C*. *elegans* model was generated by co-injection of 20 ng/μl of plasmid pPR18 (that expresses YFP in muscles) together with 30 ng/μl of Hsp72 expressed in the same tissue (plasmid pPR21) to serve as a soluble control (top panels). *C*. *elegans* injected with 20 ng/μl of h-proIAPP tagged with YFP expressed in muscles (plasmid pPR3) was used as a control for protein aggregation (middle panels). Transgenic h-proIAPP::YFP + Hsp72 *C*. *elegans* animals were generated by gonad co-injection of 20 ng/μl of plasmid pPR3 and 30 ng/μl of plasmid pPR21 (bottom panels). Transgenic animals were subjected to FRAP analysis (square). Data was collected before photobleaching (left panels), 10 seconds after photobleaching (middle panels) and 250 seconds after photobleaching (right panels). Images were obtained using an inverted confocal microscope. Results are representative of one experiment from at least three independently performed experiments with similar results. Scale bars represent 50 μm. **B.** Data represents the quantification of relative fluorescence intensity, RFI ± SEM during recovery after photobleaching of transgenic h-proIAPP::YFP *C*. *elegans* model (filled circles), transgenic h-proIAPP::YFP + Hsp72 *C*. *elegans* animals (open circles), and transgenic YFP + Hsp72 *C*. *elegans* model (filled triangles). Data are the mean of at least three independently performed experiments. *p<0.05 versus h-proIAPP::YFP, Error Bar = SEM.

To evaluate differences in solubility, we used FRAP analysis. The rate of recovery after photobleaching was used as a measure of protein solubility and allowed us to discriminate protein aggregates in which interacting proteins are immobile and insoluble. FRAP experiments on live animals that express soluble YFP + Hsp72 control, demonstrated significantly greater recovery after photobleaching (Fig 6A; top panels), while protein aggregates of transgenic h-proIAPP *C*. *elegans* animals did not recover after photobleaching, indicating the presence of immobile and insoluble aggregates (Fig 6A; middle panels). FRAP analysis of transgenic h-proIAPP + Hsp72 *C*. *elegans* demonstrated significantly increased solubility (Fig 6A; bottom panels). Quantification of relative fluorescence intensity over time provides further evidence of the ability of Hsp72 to improve h-proIAPP solubility (Fig 6B).

### ADAPT-232 improves transgenic h-proIAPP *C*. *elegans* phenotype

**Fig 7:**
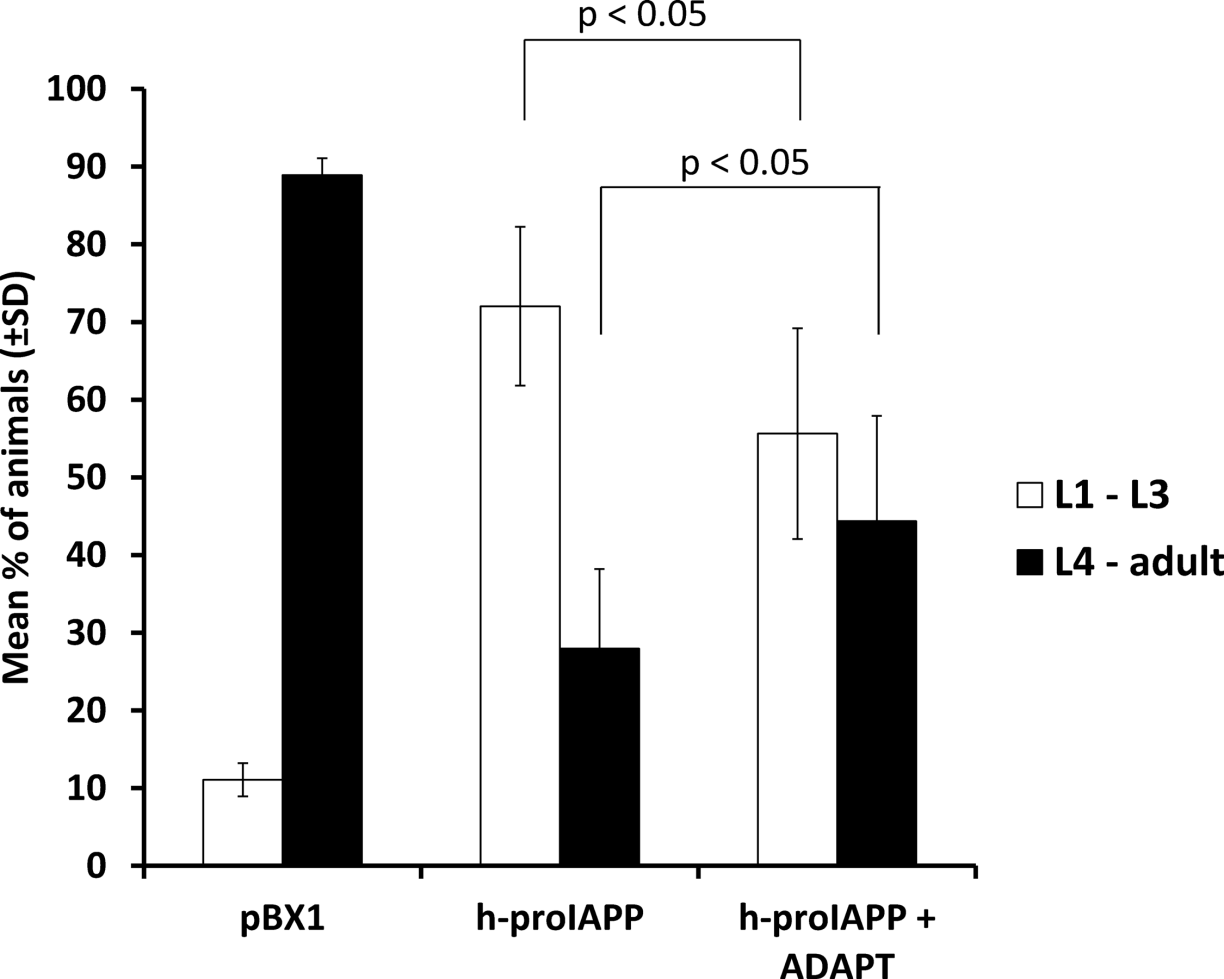
ADAPT-232 improves transgenic h-proIAPP *C*. *elegans* phenotype. Developmental phenotype was studied in the transgenic h-proIAPP *C*. *elegans* model by the evaluation of larvae or adult stages seventy-two hours after ten to fifteen gravid hermaphrodites were treated with bleach and larvae was placed in an agar plate containing 1mg/ml of ADAPT-232. Data represent the percentage of animals found at larvae 1 (L1) to larvae 3 (L3) stages (open bars) and the percentage of animals found at larvae 4 (L4) and adult stages (filled bars) ± S.D. and are the mean of three independently performed experiments. p<0.05 versus respective control.

To test the hypothesis that transgenic *C*. *elegans* expressing h-proIAPP can be used to evaluate potential drugs that decrease h-proIAPP aggregation, we used ADAPT-232 as a therapeutic candidate. ADAPT-232 was used at a concentration of 1mg/ml in NGM ADAPT-supplemented agar plates. It was previously demonstrated that the active ingredients of ADAPT-232 induced the expression of the *C*. *elegans* stress-inducible *hsp-16* [9]. We demonstrated that treatment of transgenic h-proIAPP *C*. *elegans* with ADAPT-232 significantly improved its growth retardation phenotype as compared to the untreated h-proIAPP animals (Fig 7).

## Discussion

This study was undertaken to determine the contribution of Hsp72 (HSPA1A) in preventing the toxic aggregation of h-proIAPP and h-IAPP which are the main components of intracellular amyloid [4]. We demonstrated that overexpression of Hsp72 (HSPA1A) by thermal stress or by transfection procedures protects pancreatic β-cells against exogenous and endogenous h-IAPP toxicity. Consistent with these results, we demonstrated that Hsp72 (HSPA1A) improves the solubility of h-proIAPP aggregates in a *C*. *elegans in vivo* model. Sodium azide was used at 5mM for approximately 5 to 6 minutes to immobilize the worms during imaging. Although, sodium azide is a metabolic inhibitor and a known inducer multiple heat shock proteins. The concentration and time of exposure to the worms we used in this study is not sufficient to induce heat shock proteins. This is in agreement with studies by Massie and coworkers who report heat shock protein-induction at 10 mM of sodium azide after 90 minutes exposure [21].

### Hsp72 protects pancreatic β-cells against *in vitro* h-IAPP toxicity

We demonstrated that heat shock treatment at 43°C and the overexpression of Hsp72 protect pancreatic β-cells against h-IAPP-induced toxicity (Figs 1 and 2). We propose that an important mechanism of protection involves the molecular chaperoning activity of Hsp72 that promotes folding and refolding of proteins prone to aggregate. Previous studies have demonstrated that *in vitro* Hsp72 suppresses h-IAPP misfolding, as judged by thioflavin-T fluorescence analysis [22]. We wanted to demonstrate a protein-protein interaction between Hsp72 and h-IAPP after both proteins were co-expressed in β-cells by transient transfection. However, we were not able to demonstrate such interactions either by co-immunoprecipation experiments or cross-linking procedures (data not shown). We speculated that these are transient interactions in which Hsp72 temporarily stabilizes h-IAPP and prevents its aggregation. Moreover, co-immunoprecipitation only detects proteins that remain in physiological complexes after solubilization from the cell, therefore if the complexes form large aggregates, these may not be detected. This proposed mechanism positions Hsp72 as a very promising candidate for the treatment of conformational diseases in which misfolded protein aggregates are responsible of cell toxicity.

### Toxic aggregates observed in the h-proIAPP *C*. *elegans* model correlate with growth retardation phenotype

We generated a novel transgenic *C*. *elegans* model that expresses h-proIAPP tagged with YFP for easier visualization. In these transgenic animals, a muscle promoter, pharyngeal promoter and pan-neuronal promoter were used to express a potentially secretable form of proIAPP that contained a signal-peptide sequence. This model was designed to mimic the initial fibril formation as observed in pancreatic β-cell secretory granules and to elucidate whether abnormally increased amounts of unprocessed and highly amyloidogenic proIAPP participate in the process of tissue (i.e., islet) destruction. It has been demonstrated that proIAPP is a constitutive part of the amyloid found in secretory granules by using antibodies against proIAPP NH_2_- and COOH-terminal sites [4]. Moreover, there is evidence of toxicity by IAPP oligomers in the ER of human pancreatic β-cells [23]. Given the fact that in the ER proIAPP is the predominantly existing form, it is most probable that the formation of oligomers, in this location, results from the presence of highly concentrated unprocessed proIAPP. Since all individuals secret amyloidogenic IAPP but only some of them develop type 2 diabetes, an imbalance in the proIAPP:IAPP ratio within the secretory granules may be a very important feature in the pathogenesis of the disease [24], and prolonged hyperglycemia may lead to a higher proportion of unprocessed proIAPP [25].

Biologically active peptides are synthesized as larger inactive pre-proproteins that contain an amino terminal signal peptide that is cleaved off upon entrance into the secretory pathway. This is the case of proIAPP in which prohormone convertase 2 (PC2) and prohormone convertase 1/3 (PC1/3) convert proIAPP into IAPP. Then, carboxypeptidase E removes the COOH-terminal dibasic amino acids. In *C*. *elegans*, four proprotein convertase genes were found. One of these is the *kpc-2/egl-3* gene, which encodes a PC2-like convertase that is the major active proprotein convertase in neurons [26]. In addition, carboxypeptidase E. *egl-21* gene encodes a neural-specific carboxypeptidase E [27]. It is probable that h-proIAPP in our *C*. *elegans* model is being cleaved by EGL3/KPC2 and egl-21 carboxypeptidase E, at least when expressed in neurons. Further investigation of proIAPP cleavage into a mature IAPP form, needs to be performed.

We demonstrated that h-proIAPP forms insoluble aggregates (Fig 4C) and that these aggregates correlate with a growth retardation phenotype (Fig 5). Although the physiological basis for this phenotype is not clear, it is very likely that this phenotype results from body muscle cell and pharynx muscle cell toxicity, since h-proIAPP expressed in neurons is completely secreted and accumulated in the coelomocytes. Conversely, *Drosophila Melanogaster* expressing h-proIAPP and h-IAPP have been shown to form aggregates in CNS and fat body region and flies expressing h-proIAPP in neurons showed a reduction in lifespan [28]. Toxicity can also be explained by the ability of amyloid fibrils to interact with other proteins resulting in sequestration and loss of function of the sequestered proteins [29]. Amyloid fibrils are prone to sequester components of the chaperone system [30]. Molecular chaperones, in their attempt to prevent misfolding or help with refolding activities, may get trapped within the fibrils and are prevented from exerting their chaperoning activities. This will result in the collapse of the chaperone system and propagation of folding problems along the cell. A manifestation of this toxicity may be evidenced by a development retardation phenotype exhibited by the h-proIAPP *C*. *elegans* model in which highly fluorescent protein aggregates were observed (Fig 4A; right panels and Fig 4B). Proteostasis mechanisms may not be sufficient in preventing the misfolding of the highly amyloidogenic h-proIAPP form. Conversely, protective mechanisms in the m-proIAPP *C*. *elegans* model, which expresses a protein less prone to aggregate, seem to prevent massive aggregation and a rapid clearance response was evidenced by less aggregate accumulation and reduced fluorescence intensity in their tissues (Fig 4A; left panels and Fig 4B). This is also supported by a milder developmental retardation phenotype.

### Secreted h-proIAPP is taken up by *C*. *elegans* macrophages (coelomocytes)

We were able to demonstrate that h-proIAPP expressed in neurons was secreted into the body cavity and accumulated in the coelomocytes. Adult *C*. *elegans* hermaphrodites have six fixed coelomocytes that have a phagocytic function similar to the macrophages of vertebrates (www.wormatlas.org). It seems that these coelomocytes scavenge h-proIAPP aggregates secreted from neurons in an attempt to destroy these foreign aggregates. The fact that coelomocytes in the h-proIAPP model remain highly fluorescent (Fig 4A and S3 Fig) supports the inability of these cells to destroy the aggregates. These results are in agreement with studies of cytoplasmic inclusions found in h-IAPP transgenic rat islets due to a decreased lysosomal degradation of h-IAPP toxic oligomers [31]. However, we demonstrated that not all proIAPP-expressed protein was detected extracellularly. We speculate that proIAPP deposition in pharynx and to some extent in body wall muscles may preclude extracellular secretion. Further determination of the intra- or extracellular localization of proIAPP deposits may help elucidate the fate of this peptide in muscle and pharynx cells of proIAPP transgenic animals.

### Hsp72 enhances h-proIAPP solubility and improves growth retardation phenotype in h-proIAPP *C*. *elegans* model

We demonstrated that co-expression of h-proIAPP and Hsp72 in a *C*. *elegans* model results in improved h-proIAPP solubility (Fig 6A and 6B). Similarly, the overexpression of members of the HSP70 family inhibited the formation of toxic oligomers and prevented the formation of amyloid aggregates in polyglutamine-repeat disorders, in which Hsp70 cooperates with the chaperonin TRiC [32]. Moreover, Hsp70 prevented dopaminergic neuronal loss associated with alpha-synuclein in a *Drosophila* model [33]. A genome-wide RNAi screen in an alpha-synuclein *C*. *elegans* model identified two chaperones, members of the HSP70 and HSP90 families that when knocked-down, promoted aggregation [34]. In a biochemical approach to identify chaperones that were associated with amyloid β in a *C*. *elegans* model; two members of HSP70 family were found as well as three small heat shock proteins [35]. On the basis of these findings, the pharmacological upregulation of the chaperone system gives us a new alternative to control aberrant protein misfolding and aggregation in various conformational diseases. As a proof-of-principle, some small molecules that are regulators of the heat shock response such as radicicol and geldamycin were successfully used to inhibit the formation of Huntingtin aggregates in *in vitro* studies [36]. Likewise, a group of plant extracts called adaptogens were identified for their property to reinforce a non-specific adaptive capacity to resist stress conditions and to facilitate a more rapid return to normality [9]. It was demonstrated that adaptogens induced the translocation of the transcription factor DAF-16 in *C*. *elegans* (FOXO in humans) into the nucleus [9]. Several genes regulated by DAF-16 are involved in stress resistance, longevity and autophagy [37], a process required for the removal of protein aggregates. Moreover, adaptogens increased the synthesis of the *C*. *elegans* stress-inducible Hsp-16 (Wiegant et al. 2009). Therefore, adaptogens may target two important mechanisms of cell protection against toxic aggregates, a) by increasing the levels of molecular chaperones, which prevent the formation of aggregates, and b) by promoting the autophagic degradation of toxic aggregates.

Adaptogens were successfully used in animal models of aging related disorders and conformational diseases. Panossian et al., demonstrated that ADAPT-232 increased Hsp72 serum levels in mice and protected them against stress (Panossian, et al. 2009). Moreover, *Eleutherococcus senticocus* and *Rhodiola rosea* prolonged the lifespan of N2 wild-type *C*. *elegans* strain in a dose-dependent manner and increased stress resistance against acute and chronic heat stress and against chronic oxidative stress conditions (Wiegant et al. 2009). The same effect was evidenced in *Drosophila melanogaster*, where *Rhodiola rosea* showed to increase its lifespan [38]. Bai et al., demonstrated that *Eleutherococcus senticocus* extracts promotes the regeneration of neuritic atrophy and synaptic loss caused by amyloid β in rat cultured cortical neurons [39]. In this study, we demonstrated that plant adaptogens (ADAPT-232) were able to improve h-proIAPP *C*. *elegans* phenotype (Fig 7). Taken together, these results suggest that adaptogens may be used as a potential drug to treat type 2 diabetes. Other heat shock response modulators, previously used for other protein conformational diseases, including curcumin, geldanamycin and radicicol [40, 41] should be studied for their efficacy in this model.

In conclusion, our *in vitro* studies show that Hsp72 (HSPA1A) ameliorates exogenous and endogenous h-IAPP toxicity. Thus, interventions designed to increase the levels of Hsp72 in pancreatic β-cells may constitute potential treatments for type 2 diabetes. The *C*. *elegans* model described in this study does not replicate all aspects of type 2 diabetes, but it captures a very important mechanism of pancreatic β-cell toxicity mediated by h-proIAPP aggregation. Additionally, this model allows researchers a fast and effective technique to investigate factors and potential drugs than can modulate h-proIAPP aggregation.

## Supporting Information

S1 File Supporting Information. (DOCX)

S1 Table Primers used in current study. (DOCX)

S1 Fig DS-Red::h-IAPP transfection in Beta-TC6 cells. Cells that exhibited strongest red fluorescence were round and detached **A.** Fluorescence image (left), phase contrast image (middle), fluorescence and phase contrast overlaid image (right), using 40 X magnifications, 24 hours after transfection. **B.** Fluorescence image (left), phase contrast image (middle), fluorescence and phase contrast overlaid image (right), using 40 X magnifications, 48 hours after transfection.
(TIF)

S2 Fig Identification of positive proIAPP clones by colony PCR. Top panel; lane 1, 1-kb DNA ladder; lane 2–11, DNA extracted from various h-proIAPP colonies; lane 12, h-proIAPP vector (Origene) as a positive control. Bottom panel; lane 1–10, DNA extracted from different m-proIAPP colonies; lane 11, pCMV6 (Origene) as a negative control; lane 12, 1-kb DNA ladder.
(TIF)

S3 Fig Coelomocytes in the h-proIAPP transgenic *C*. *elegans* model. **A.** h-proIAPP adult *C*. *elegans* ventral coelomocytes and **B.** Dorsal coelomocytes images obtained with a fluorescent microscope at 40X magnification. Coelomocytes are suggested to be phagocytic and similar in function to the macrophages of vertebrates. They have a relatively fixed position in the body cavity. **C.** h-proIAPP coelomocytes fluorescence images and **D.** Phase contrast images using 100X magnification.
(TIF)

S4 Fig Gene transcript level in h-proIAPP and m-proIAPP *C*. *elegans* models. RT-PCR was performed on h-proIAPP and m-proIAPP worms and PCR samples were taken at 25, 27 and 29 cycles of amplification and semi-quantified by visualization following electrophoresis in 1.5% agarose gels. Lane 1, 1-kb DNA ladder; lane 2, PCR product taken at 25 cycles of amplification of m-proIAPP gene; lane 3, PCR product taken at 25 cycles of amplification of h-proIAPP gene; lane 4, PCR product taken at 27 cycles of amplification of m-proIAPP gene; lane 5, PCR product taken at 27 cycles of amplification of h-proIAPP gene; lane 6, empty; lane 7, PCR product taken at 29 cycles of amplification of m-proIAPP gene; lane 8, PCR product taken at 29 cycles of amplification of h-proIAPP gene.
(TIF)

S5 Fig Acute inducible expression of h-proIAPP in a *C*. *elegans* model driven by *hsp-16-2* promoter, correlates with high number of aggregates. Mouse and human pro-IAPP tagged with YFP expressed under the inducible *hsp-16-2* promoter were observed in **A**, pharynx; **B**, body wall muscles; **C**, intestine, after animals were exposed to heat stress at 33°C for 90 minutes. Images were obtained using a fluorescent microscope at 40X magnification. Arrows indicate areas of aggregation.
(TIFF)

## References

[1] Hull RL, Westermark GT, Westermark P, Kahn SE. Islet amyloid: a critical entity in the pathogenesis of type 2 diabetes. J Clin Endocrinol Metab. 2004;89(8):3629–43. Epub 2004/08/05. 10.1210/jc.2004-0405 . 15292279

[2] Campbell RK. Fate of the beta-cell in the pathophysiology of type 2 diabetes. Journal of the American Pharmacists Association: JAPhA. 2009;49 Suppl 1:S10–5. Epub 2009/10/06. 10.1331/JAPhA.2009.09076 . 19801360

[3] Westermark P, Andersson A, Westermark GT. Islet amyloid polypeptide, islet amyloid, and diabetes mellitus. Physiol Rev. 2011;91(3):795–826. Epub 2011/07/12. 10.1152/physrev.00042.2009 . 21742788

[4] Paulsson JF, Andersson A, Westermark P, Westermark GT. Intracellular amyloid-like deposits contain unprocessed pro-islet amyloid polypeptide (proIAPP) in beta cells of transgenic mice overexpressing the gene for human IAPP and transplanted human islets. Diabetologia. 2006;49(6):1237–46. Epub 2006/03/30. 10.1007/s00125-006-0206-7 . 16570161

[5] Paulsson JF, Westermark GT. Aberrant processing of human proislet amyloid polypeptide results in increased amyloid formation. Diabetes. 2005;54(7):2117–25. Epub 2005/06/29. . 15983213 10.2337/diabetes.54.7.2117

[6] Krampert M, Bernhagen J, Schmucker J, Horn A, Schmauder A, Brunner H, et al Amyloidogenicity of recombinant human pro-islet amyloid polypeptide (ProIAPP). Chem Biol. 2000;7(11):855–71. Epub 2000/11/30. . 11094339 10.1016/s1074-5521(00)00034-x

[7] Hartl FU, Bracher A, Hayer-Hartl M. Molecular chaperones in protein folding and proteostasis. Nature. 2011;475(7356):324–32. Epub 2011/07/22. 10.1038/nature10317 . 21776078

[8] Kettern N, Dreiseidler M, Tawo R, Hohfeld J. Chaperone-assisted degradation: multiple paths to destruction. Biol Chem. 2010;391(5):481–9. Epub 2010/03/23. 10.1515/BC.2010.058 . 20302520

[9] Wiegant FA, Surinova S, Ytsma E, Langelaar-Makkinje M, Wikman G, Post JA. Plant adaptogens increase lifespan and stress resistance in C. elegans. Biogerontology. 2009;10(1):27–42. Epub 2008/06/10. 10.1007/s10522-008-9151-9 . 18536978

[10] Panossian A, Wikman G, Kaur P, Asea A. Adaptogens exert a stress-protective effect by modulation of expression of molecular chaperones. Phytomedicine: international journal of phytotherapy and phytopharmacology. 2009;16(6–7):617–22. Epub 2009/02/04. 10.1016/j.phymed.2008.12.003 . 19188053

[11] Prahlad V, Morimoto RI. Neuronal circuitry regulates the response of Caenorhabditis elegans to misfolded proteins. Proc Natl Acad Sci U S A. 2011;108(34):14204–9. Epub 2011/08/17. 10.1073/pnas.1106557108 21844355 PMC3161566

[12] Schnabel H, Schnabel R. An Organ-Specific Differentiation Gene, pha-1, from Caenorhabditis elegans. Science. 1990;250(4981):686–8. Epub 1990/11/02. 10.1126/science.250.4981.686 . 17810870

[13] Hodgkin J, Horvitz HR, Brenner S. Nondisjunction Mutants of the Nematode CAENORHABDITIS ELEGANS. Genetics. 1979;91(1):67–94. Epub 1979/01/01. 17248881 10.1093/genetics/91.1.67 PMC1213932

[14] Edwards SL, Charlie NK, Milfort MC, Brown BS, Gravlin CN, Knecht JE, et al A novel molecular solution for ultraviolet light detection in Caenorhabditis elegans. PLoS Biol. 2008;6(8):e198 Epub 2008/08/09. 10.1371/journal.pbio.0060198 18687026 PMC2494560

[15] Brenner S. The genetics of Caenorhabditis elegans. Genetics. 1974;77(1):71–94. Epub 1974/05/01. 4366476 10.1093/genetics/77.1.71 PMC1213120

[16] Mello CC, Kramer JM, Stinchcomb D, Ambros V. Efficient gene transfer in C.elegans: extrachromosomal maintenance and integration of transforming sequences. EMBO J. 1991;10(12):3959–70. Epub 1991/12/01. 1935914 10.1002/j.1460-2075.1991.tb04966.x PMC453137

[17] Granato M, Schnabel H, Schnabel R. pha-1, a selectable marker for gene transfer in C. elegans. Nucleic Acids Res. 1994;22(9):1762–3. Epub 1994/05/11. 8202383 10.1093/nar/22.9.1762 PMC308061

[18] Brignull HR, Moore FE, Tang SJ, Morimoto RI. Polyglutamine proteins at the pathogenic threshold display neuron-specific aggregation in a pan-neuronal Caenorhabditis elegans model. J Neurosci. 2006;26(29):7597–606. Epub 2006/07/21. 10.1523/JNEUROSCI.0990-06.2006 . 16855087 PMC6674286

[19] Phair RD, Misteli T. High mobility of proteins in the mammalian cell nucleus. Nature. 2000;404(6778):604–9. Epub 2000/04/15. 10.1038/35007077 . 10766243

[20] Lorenzo A, Razzaboni B, Weir GC, Yankner BA. Pancreatic islet cell toxicity of amylin associated with type-2 diabetes mellitus. Nature. 1994;368(6473):756–60. Epub 1994/04/21. 10.1038/368756a0 . 8152488

[21] Massie MR, Lapoczka EM, Boggs KD, Stine KE, White GE. Exposure to the metabolic inhibitor sodium azide induces stress protein expression and thermotolerance in the nematode Caenorhabditis elegans. Cell Stress Chaperones. 2003;8(1):1–7. 12820649 10.1379/1466-1268(2003)8<1:ettmis>2.0.co;2 PMC514849

[22] Chien V, Aitken JF, Zhang S, Buchanan CM, Hickey A, Brittain T, et al The chaperone proteins HSP70, HSP40/DnaJ and GRP78/BiP suppress misfolding and formation of beta-sheet-containing aggregates by human amylin: a potential role for defective chaperone biology in Type 2 diabetes. Biochem J. 2010;432(1):113–21. Epub 2010/08/26. 10.1042/BJ20100434 . 20735358

[23] Gurlo T, Ryazantsev S, Huang CJ, Yeh MW, Reber HA, Hines OJ, et al Evidence for proteotoxicity in beta cells in type 2 diabetes: toxic islet amyloid polypeptide oligomers form intracellularly in the secretory pathway. Am J Pathol. 176(2):861–9. 10.2353/ajpath.2010.090532 20042670 PMC2808091

[24] Zheng X, Ren W, Zhang S, Liu J, Li S, Li J, et al Serum levels of proamylin and amylin in normal subjects and patients with impaired glucose regulation and type 2 diabetes mellitus. Acta diabetologica. 2010;47(3):265–70. Epub 2010/05/29. 10.1007/s00592-010-0201-9 . 20509034

[25] Hou X, Ling Z, Quartier E, Foriers A, Schuit F, Pipeleers D, et al Prolonged exposure of pancreatic beta cells to raised glucose concentrations results in increased cellular content of islet amyloid polypeptide precursors. Diabetologia. 1999;42(2):188–94. Epub 1999/03/04. 10.1007/s001250051138 . 10064099

[26] Kass J, Jacob TC, Kim P, Kaplan JM. The EGL-3 proprotein convertase regulates mechanosensory responses of Caenorhabditis elegans. J Neurosci. 2001;21(23):9265–72. Epub 2001/11/22. . 11717360 10.1523/JNEUROSCI.21-23-09265.2001 PMC6763909

[27] Jacob TC, Kaplan JM. The EGL-21 carboxypeptidase E facilitates acetylcholine release at Caenorhabditis elegans neuromuscular junctions. J Neurosci. 2003;23(6):2122–30. Epub 2003/03/27. . 12657671 10.1523/JNEUROSCI.23-06-02122.2003 PMC6742027

[28] Schultz SW, Nilsson KP, Westermark GT. Drosophila melanogaster as a model system for studies of islet amyloid polypeptide aggregation. PLoS One. 2011;6(6):e20221 Epub 2011/06/23. 10.1371/journal.pone.0020221 21695120 PMC3114789

[29] Olzscha H, Schermann SM, Woerner AC, Pinkert S, Hecht MH, Tartaglia GG, et al Amyloid-like aggregates sequester numerous metastable proteins with essential cellular functions. Cell. 2011;144(1):67–78. Epub 2011/01/11. 10.1016/j.cell.2010.11.050 . 21215370

[30] Balch WE, Morimoto RI, Dillin A, Kelly JW. Adapting proteostasis for disease intervention. Science. 2008;319(5865):916–9. Epub 2008/02/16. 10.1126/science.1141448 . 18276881

[31] Rivera JF, Gurlo T, Daval M, Huang CJ, Matveyenko AV, Butler PC, et al Human-IAPP disrupts the autophagy/lysosomal pathway in pancreatic beta-cells: protective role of p62-positive cytoplasmic inclusions. Cell Death Differ. 2011;18(3):415–26. Epub 2010/09/04. 10.1038/cdd.2010.111 20814419 PMC3132000

[32] Behrends C, Langer CA, Boteva R, Bottcher UM, Stemp MJ, Schaffar G, et al Chaperonin TRiC promotes the assembly of polyQ expansion proteins into nontoxic oligomers. Mol Cell. 2006;23(6):887–97. Epub 2006/09/16. 10.1016/j.molcel.2006.08.017 . 16973440

[33] Auluck PK, Chan HY, Trojanowski JQ, Lee VM, Bonini NM. Chaperone suppression of alpha-synuclein toxicity in a Drosophila model for Parkinson's disease. Science. 2002;295(5556):865–8. Epub 2002/02/02. 10.1126/science.1067389 . 11823645

[34] Roodveldt C, Bertoncini CW, Andersson A, van der Goot AT, Hsu ST, Fernandez-Montesinos R, et al Chaperone proteostasis in Parkinson's disease: stabilization of the Hsp70/alpha-synuclein complex by Hip. EMBO J. 2009;28(23):3758–70. Epub 2009/10/31. 10.1038/emboj.2009.298 19875982 PMC2790486

[35] Fonte V, Kapulkin V, Taft A, Fluet A, Friedman D, Link CD. Interaction of intracellular beta amyloid peptide with chaperone proteins. Proc Natl Acad Sci U S A. 2002;99(14):9439–44. Epub 2002/06/29. 10.1073/pnas.152313999 12089340 PMC123159

[36] Hay DG, Sathasivam K, Tobaben S, Stahl B, Marber M, Mestril R, et al Progressive decrease in chaperone protein levels in a mouse model of Huntington's disease and induction of stress proteins as a therapeutic approach. Hum Mol Genet. 2004;13(13):1389–405. Epub 2004/04/30. 10.1093/hmg/ddh144 . 15115766

[37] Demontis F, Perrimon N. FOXO/4E-BP signaling in Drosophila muscles regulates organism-wide proteostasis during aging. Cell. 2010;143(5):813–25. Epub 2010/11/30. 10.1016/j.cell.2010.10.007 21111239 PMC3066043

[38] Jafari M, Felgner JS, Bussel II, Hutchili T, Khodayari B, Rose MR, et al Rhodiola: a promising anti-aging Chinese herb. Rejuvenation research. 2007;10(4):587–602. Epub 2007/11/10. 10.1089/rej.2007.0560 . 17990971

[39] Bai Y, Tohda C, Zhu S, Hattori M, Komatsu K. Active components from Siberian ginseng (Eleutherococcus senticosus) for protection of amyloid beta(25–35)-induced neuritic atrophy in cultured rat cortical neurons. Journal of natural medicines. 2011;65(3–4):417–23. Epub 2011/02/09. 10.1007/s11418-011-0509-y . 21301979

[40] Westerheide SD, Morimoto RI. Heat shock response modulators as therapeutic tools for diseases of protein conformation. J Biol Chem. 2005;280(39):33097–100. Epub 2005/08/04. 10.1074/jbc.R500010200 . 16076838

[41] Daval M, Bedrood S, Gurlo T, Huang CJ, Costes S, Butler PC, et al The effect of curcumin on human islet amyloid polypeptide misfolding and toxicity. Amyloid: the international journal of experimental and clinical investigation: the official journal of the International Society of Amyloidosis. 2010;17(3–4):118–28. Epub 2010/11/12. 10.3109/13506129.2010.530008 . 21067307 PMC4394664

