# Supplementary material for: Hsp72 (HSPA1A) Prevents Human Islet Amyloid Polypeptide Aggregation and Toxicity: A New Approach for Type 2 Diabetes Treatment
Source: PLoS One. 2016 Mar 9;11(3):e0149409. doi: 10.1371/journal.pone.0149409 (PMC4784952; doi:10.1371/journal.pone.0149409)
Supplement: S1 File — (DOCX) [file pone.0149409.s001.docx]

**S1 File**

**Supplemental Methods**

**Nematode culture and maintenance**

NGM plates were prepared using 3 g NaCl, 2.5 g Bacto-peptone, 17 g Bacto-agar, 1 ml cholesterol in ethanol solution (5 mg/ml), 1 ml 1M MgSO_4_, 1 ml 1M CaCl_2_, 25 ml 1M KH_2_PO_4_ (pH 6.0) [[1](#_ENREF_1)] and 1 ml Nystatin in ethanol solution (200 mg Nystatin/14 ml 95% ethanol) in 1L dH_2_0. NGM ADAPT-supplemented plates were prepared by adding adaptogen extracts, ADAPT-232 forte, is a proprietary name of a fixed combination of three genuine (native) extracts of Eleutherococcus senticocus (Rupr. Et Maxim) Harms root, Schisandra chinensis (Turzc) Baill root, Rhodiola rosea L. root and vitamin B5, characterized for the content of eleutherosides E and B (0.17%), schisandrin and gamma-schisandrin (0.85%), salidroside (0.33%), tirosol (0.07%), rosavin (0.37%), triandrin (0.01%) and calcium panthotenate (42.8%); kindly provided by Dr. A. Panossian from the Swedish Herbal Institute [[2](#_ENREF_2)]. The extracts were freshly dissolved in sterile water to prepare a stock solution at a concentration of 100 mg/ml. To supplement the agar plates with plant adaptogen, the solution was added during the preparatory process of the agar at a concentration of 1 mg/ml. Bleaching solution contained 100 μl 4N NaOH and 150 μl 5.25% NaOCl. M9 Buffer was composed of 6 g Na_2_HPO_4_, 3 g KH_2_PO_4_, 5 g NaCl in 1 L dH_2_O. Before using, 1000 μl of 1M MgSO_4_ was added.

*C. elegans* were maintained on NGM plates seeded with *Escherichia coli* (strain OP50) as food source at 20°C. Once the animals depleted the bacteria, a piece of agar from the old plate was transferred to the new plate seeded with OP50. Or else animals were picked under the microscope and transferred to the new plate.

**Generation of transgenics**

Plasmids containing the construct of interest were injected into 1-day-old adult pha-1(e2123 ); him-5(e1490); lite-1(ce314) hermaphrodites following standard procedures [[3](#_ENREF_3)]. Briefly, healthy young adult hermaphrodites, with visible gonads, were transferred to a 2% agarose pad in order to immobilize them for the procedure. Injection needles were produced from thin glass capillaries and loaded with DNA solution. A high resolution inverted microscope that counts with a mechanical micromanipulator that holds the needle and bring it into the correct position for injection was used. Microinjection oil is used to prevent the worm dehydration while it lies in the agarose layer. Using 40X objective the cyncytial gonad arm was focused and the worm is gently moved toward the needle for injection of DNA solution (gas pressure goes through the needle at around 20 psi to push DNA solution). Once injected the worm is moved away from the needle and transferred into a drop of M9 solution on a new OP50 seeded plate in order to wash the excess of oil. The pha-1 rescuing plasmid, pBX1 (50 ng/μl), was used as a transgenic marker [[4](#_ENREF_4)]. pha-1 is a temperature-sensitive embryonic lethal mutation, which allows worms to grow normally at 15°C; however, at 20°C the mutation is 100% embryonic lethal [[5](#_ENREF_5)]. Thus, transgenic animals that carry pBX1 (pha-1 rescue) are selected due to their ability to grow at 20°C. pUC18 was used as carrier DNA to bring each injection mixture to a final concentration of 200 ng/μl. For each plasmid injected, concentrations were as follows: 20 ng/μl was used for pPR3, pPR4, pPR5, pPR8, pPR9, pPR10 and pPR18; and 30 ng/μl was used for pPR21. Once stable transgenic lines were obtained, hermaphrodites from two or more independent transgenic lines were scored for the developmental retardation phenotype as described below. Transgenics were also studied using fluorescent and confocal microscopy.

**Characterization of *C. elegans* larvae to adult life cycle**

*C. elegans* nematodes have two sexes: a self-fertilizing hermaphrodite (XX) and the male (X0). The frequency of the male is very low ([www.wormatlas.org](http://www.wormatlas.org)). Self-fertile hermaphrodites produce sperm which is stored in the spermathecal and produce oocytes that are fertilized by the sperm in the first day of the L4/adult hermaphrodite ([www.wormatlas.org](http://www.wormatlas.org)). *C. elegans* life cycle comprises the embryonic stage, four larvae stages (L1-L4) and the adult stage. Larvae and adult stages were identified by visualization under the microscope. Animals increase in size throughout the four larval stages (L1-L4). Therefore, L1 stage larvae are the smallest and L2 and L3 are bigger, but all with similar appearance. At the L4 stage, hermaphrodites have a tapered tail and the developing vulva can be seen as a clear half circle in the center of the ventral side. The males have a wider tail. In adults, the hermaphrodites have a wider girth and tapered tail and the males show a slimmer girth and fan-shaped tail ([www.wormbook.org](http://www.wormbook.org)).

**Fluorescence recovery after photobleaching (FRAP) analysis**

FRAP analysis was performed using an Olympus Fluoview 300 confocal microscope with 60X apochromat water immersion objective. FRAP was used to determine the biophysical properties of IAPP aggregates and was performed on 1-day-old animals. Imaging was at 10% power of a 488 nm laser and bleaching was performed by 5 interactions at 100% power [[6](#_ENREF_6)]. Relative fluorescence intensity, RFI, was determined as: (T_t_/C_t_)/(T_0_/C_0_) where T_0_ represents the total intensity of the region of interest before photobleaching and T_t_ is the intensity of the same area at time point t. We normalized against an unbleached area, where C_0_ is a control area before bleaching and C_t_ is the same area at time point t after bleaching [[6](#_ENREF_6), [7](#_ENREF_7)]. Images were obtained using the Olympus Fluoview 300 confocal microscope and analyzed using ImageJ.

***C. elegans* total RNA extraction and RT-PCR**

Worms were grown on NGM plates containing OP50 and were harvested before starvation with M9 buffer in 1.5-ml microcentrifuge tubes. Worms were collected by centrifugation at 1000 rpm and the supernatant was removed. Zirconium oxide beads and 1 ml of TRIzol (Total RNA Isolation) reagent (Life Technologies) were added to the worm mass. A shaker was used to grind up the worms. Then tubes were centrifuged at 13,000 rpm for 10 min at 4°C. Supernatant was removed and transferred into an RNAse free microcentrifuge tube; 200 μl of chloroform was added and the mixture was vortexed until an emulsion was formed. Tubes were centrifuged at 13,000 rpm for 15 min at 4°C. The aqueous top layer that contains RNA was carefully removed and transferred into another RNAse free microcentrifuge tube and 500 μl of isopropanolol was added to precipitate RNA. Tubes were centrifuged at 13,000 rpm for 10 min at 4°C and the supernatant was carefully removed and pellet washed with 100 μl of 75% ethanol. Tubes were centrifuged again at 13,000 rpm for 5 min at 4°C, the supernatant was discarded and the pellet was allowed to dry for about 10 min. and then dissolved with 20 μl of DEPC-treated water. The quantity of RNA was measured using a nano-spectrophotometer. cDNA synthesis was performed using 1 μg of total RNA from each sample with the SuperScriptTM III First-Strand Synthesis System for RT-PCR kit (Life Technologies). PCR was performed using forward (5′-CGACTCTAGAGGATCCATGGGCATCCTGAAGCTGCAAG-3′) and reverse (5′-CCAATCCCGGGGATCCAAGGGGCAAGTAATTCAGTGG-3′) primers for h-proIAPP cloning and forward (5′-CGACTCTAGAGGATCCATGATGTGCATCTCCAAACTGCCAGC-3′) and reverse (5′-CCAATCCCGGGGATCCAACGAGTAAGAAATCCAAGG-3′) primers for m-proIAPP cloning. PCR samples were taken at 25, 27 and 29 cycles of amplification and semi-quantified by 1.5% agarose gel electrophoresis. Image processor Quantity One (Version 4.6) was used to scan and to compare expression levels.

**Supplemental Results**

**Fluorescent h-IAPP aggregates in Beta-TC-6 cells**

In order to determine the localization of h-IAPP aggregates in transfected Beta-TC-6 cells, we created a DsRed-hIAPP plasmid using the DsRed-Monomer-C1 Vector 4.7Kb (Clontech). h-IAPP was inserted using Sal1 restriction site located at the C-terminus of DsRed-Monomer. Beta-TC-6 cells were transfected with Ds-Red-hIAPP using Lipofectamine LTX and PLUS reagent (Life Technologies). Medium was changed after 4-6 hours and cells were incubated for 24 to 48 hours. Standard fluorescence microscopy was performed using an Olympus CKX41 microscope. A DP71 CCD camera was used to capture phase contrast and fluorescence images with DP71 image acquisition interface software (Olympus). The fluorescence intensity of cells expressing DsRed-h-IAPP showed great variation. Cells that exhibited the strongest red fluorescence were round and detached suggesting that they were dead due to h-IAPP toxicity (S1 Fig). Therefore, we could not image live cells to determine the localization of amylin aggregates. An additional problem we encountered was the size of the Ds-Red monomer-C1. The size of this red fluorescent protein is 5 to 6 times larger than h-IAPP; thus, the entire structure may be more prone to aggregate and for that reason may be more toxic.

**Inducible h-proIAPP expression correlates with higher number of aggregates**

To determine the effect of acute expression of proIAPP on *C. elegans*, we used an inducible system in which we recombined pNG1 and pNG2 with pBL172 (*hsp-16-2* promoter) using LR clonase (Life Technologies) to create h-proIAPP plasmid, pPR2 and m-proIAPP plasmid, pPR7. The pPR2 and pPR7 plasmids were independently injected at a concentration of 9 ng/μl along with 50 ng/μl marker plasmid pBX1 to create transgenic C*. elegans* strains in which the transcription of h-proIAPP::tagYFP and m-proIAPP::tagYFP were driven by the *hsp-16-2* gene promoter. In this fashion, we expressed h- and m-proIAPP in an acute, inducible way in response to heat stress. Plasmids expressed under the inducible hsp-16-2 promoter produced strong pharynx, body wall muscles and intestinal proIAPP expression after animals were exposed to heat stress at 33°C for 90 minutes (S5 Fig). Transgenic h-proIAPP *C. elegans* animals exhibited significantly more aggregates than transgenic m-proIAPP *C. elegans* animals (S5 Fig; right panels), especially in pharynx. Interestingly, transgenic worms were able to restore proteostasis and get rid of protein aggregates as no fluorescence was detected 48 hours post heat stress treatment (data not shown). These results suggest that transient expression of h-proIAPP driven by a hsp-16-2 promoter does not overload the *C. elegans* proteostasis mechanisms, such as autophagy. Importantly, heat stress also induces the worm endogenous expression of hsp-16-2 that stimulates the autophagic degradation of h-proIAPP aggregates.

**Supplemental References**

1. Brenner S. The genetics of Caenorhabditis elegans. Genetics. 1974;77(1):71-94. Epub 1974/05/01. PubMed PMID: 4366476; PubMed Central PMCID: PMC1213120.

2. Panossian A, Wikman G, Kaur P, Asea A. Adaptogens exert a stress-protective effect by modulation of expression of molecular chaperones. Phytomedicine : international journal of phytotherapy and phytopharmacology. 2009;16(6-7):617-22. PubMed PMID: 19188053.

3. Mello CC, Kramer JM, Stinchcomb D, Ambros V. Efficient gene transfer in C.elegans: extrachromosomal maintenance and integration of transforming sequences. EMBO J. 1991;10(12):3959-70. Epub 1991/12/01. PubMed PMID: 1935914; PubMed Central PMCID: PMC453137.

4. Granato M, Schnabel H, Schnabel R. pha-1, a selectable marker for gene transfer in C. elegans. Nucleic Acids Res. 1994;22(9):1762-3. Epub 1994/05/11. PubMed PMID: 8202383; PubMed Central PMCID: PMC308061.

5. Schnabel H, Schnabel R. An Organ-Specific Differentiation Gene, pha-1, from Caenorhabditis elegans. Science. 1990;250(4981):686-8. Epub 1990/11/02. doi: 10.1126/science.250.4981.686. PubMed PMID: 17810870.

6. Brignull HR, Moore FE, Tang SJ, Morimoto RI. Polyglutamine proteins at the pathogenic threshold display neuron-specific aggregation in a pan-neuronal Caenorhabditis elegans model. J Neurosci. 2006;26(29):7597-606. Epub 2006/07/21. doi: 10.1523/JNEUROSCI.0990-06.2006. PubMed PMID: 16855087.

7. Phair RD, Misteli T. High mobility of proteins in the mammalian cell nucleus. Nature. 2000;404(6778):604-9. Epub 2000/04/15. doi: 10.1038/35007077. PubMed PMID: 10766243.
