# Supplementary material for: Hsp72 (HSPA1A) Prevents Human Islet Amyloid Polypeptide Aggregation and Toxicity: A New Approach for Type 2 Diabetes Treatment
Source: PLoS One. 2016 Mar 9;11(3):e0149409. doi: 10.1371/journal.pone.0149409 (PMC4784952; doi:10.1371/journal.pone.0149409)
Supplement: S1 Table — (DOCX) [file pone.0149409.s002.docx]

**S1 Table. Primers used in current study.**

| **Plasmid** | **Contents** |
| --- | --- |
| pBL172 | Gateway entry clone containing *hsp-16-2* promoter |
| pLR22 | Gateway entry clone containing *lev-11* muscle-specific promoter |
| pLR25 | Gateway entry clone containing *tnt-4* pharyngeal-specific promoter |
| pLR35 | Gateway entry clone containing *aex-3* pan-neuronal promoter |
| pNG1 | Gateway RFC C.1:h-proIAPP:YFP |
| pNG2 | Gateway RFC C.1:m-proIAPP:YFP |
| pNG3 | Gateway RFC C.1:YFP |
| pNG4 | Gateway RFC C.1:Hsp72 |
| pPR2 | P_hsp-16-2_:h-proIAPP:YFP |
| pPR3 | P_lev-11_:h-proIAPP:YFP |
| pPR4 | P_tnt-4_:h-proIAPP:YFP |
| pPR5 | P_aex-3_:h-proIAPP:YFP |
| pPR7 | P_hsp-16-2_:m-proIAPP:YFP |
| pPR8 | P_lev-11_:m-proIAPP:YFP |
| pPR9 | P_tnt-4_:m-proIAPP:YFP |
| pPR10 | P_aex-3_:m-proIAPP:YFP |
| pPR18 | P_lev-11_:YFP |
| pPR21 | P_lev-11_:Hsp72 |
