# Supplementary figures and images for: Hsp72 (HSPA1A) Prevents Human Islet Amyloid Polypeptide Aggregation and Toxicity: A New Approach for Type 2 Diabetes Treatment
Source: PLoS One. 2016 Mar 9;11(3):e0149409. doi: 10.1371/journal.pone.0149409 (PMC4784952; doi:10.1371/journal.pone.0149409)

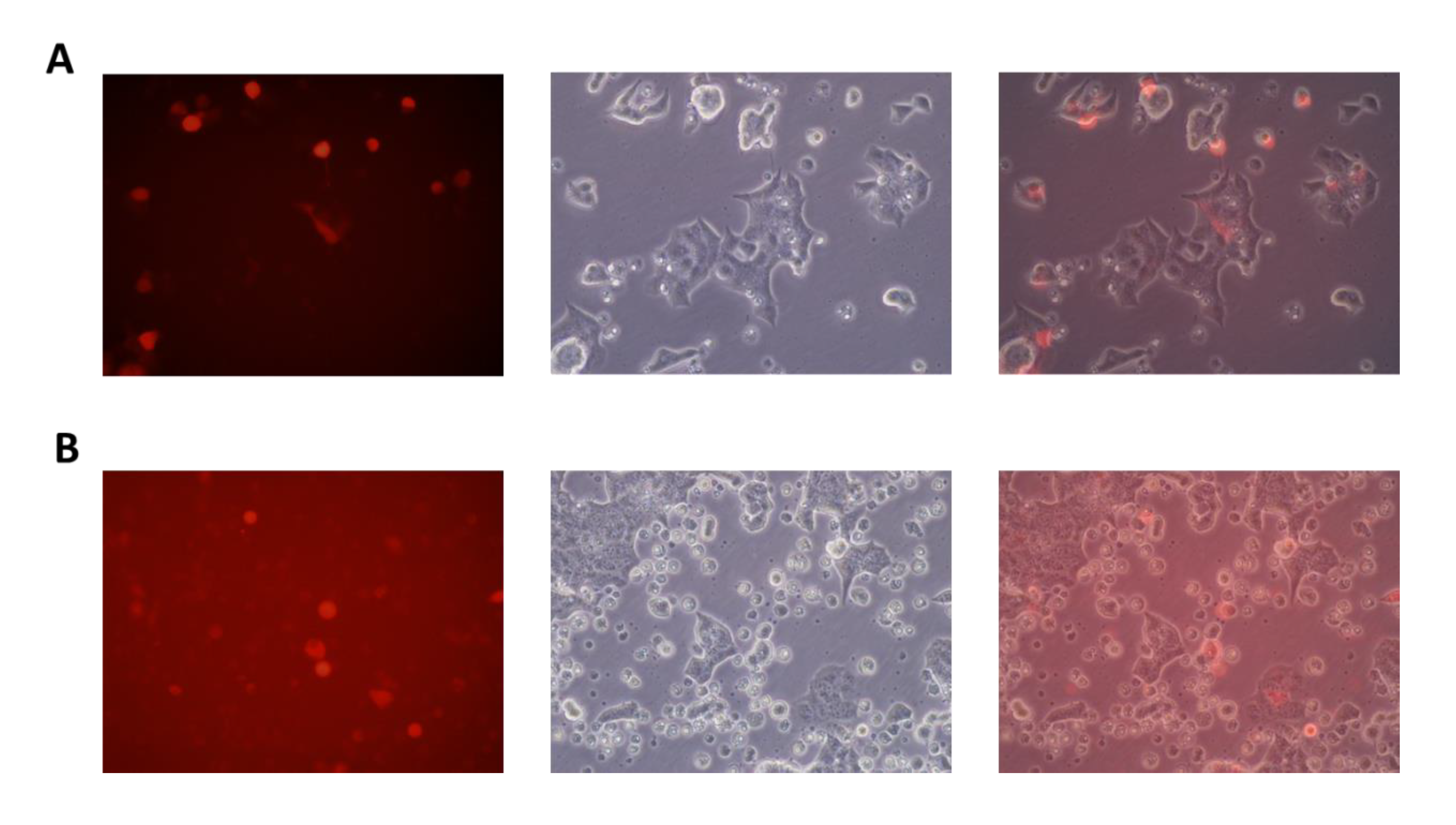

Supplement: S1 Fig — Cells that exhibited strongest red fluorescence were round and detached A. Fluorescence image (left), phase contrast image (middle), fluorescence and phase contrast overlaid image (right), using 40 X magnifications, 24 hours after transfection. B. Fluorescence image (left), phase contrast image (middle), fluorescence and phase contrast overlaid image (right), using 40 X magnifications, 48 hours after transfection. (TIF) [file pone.0149409.s003.tif]

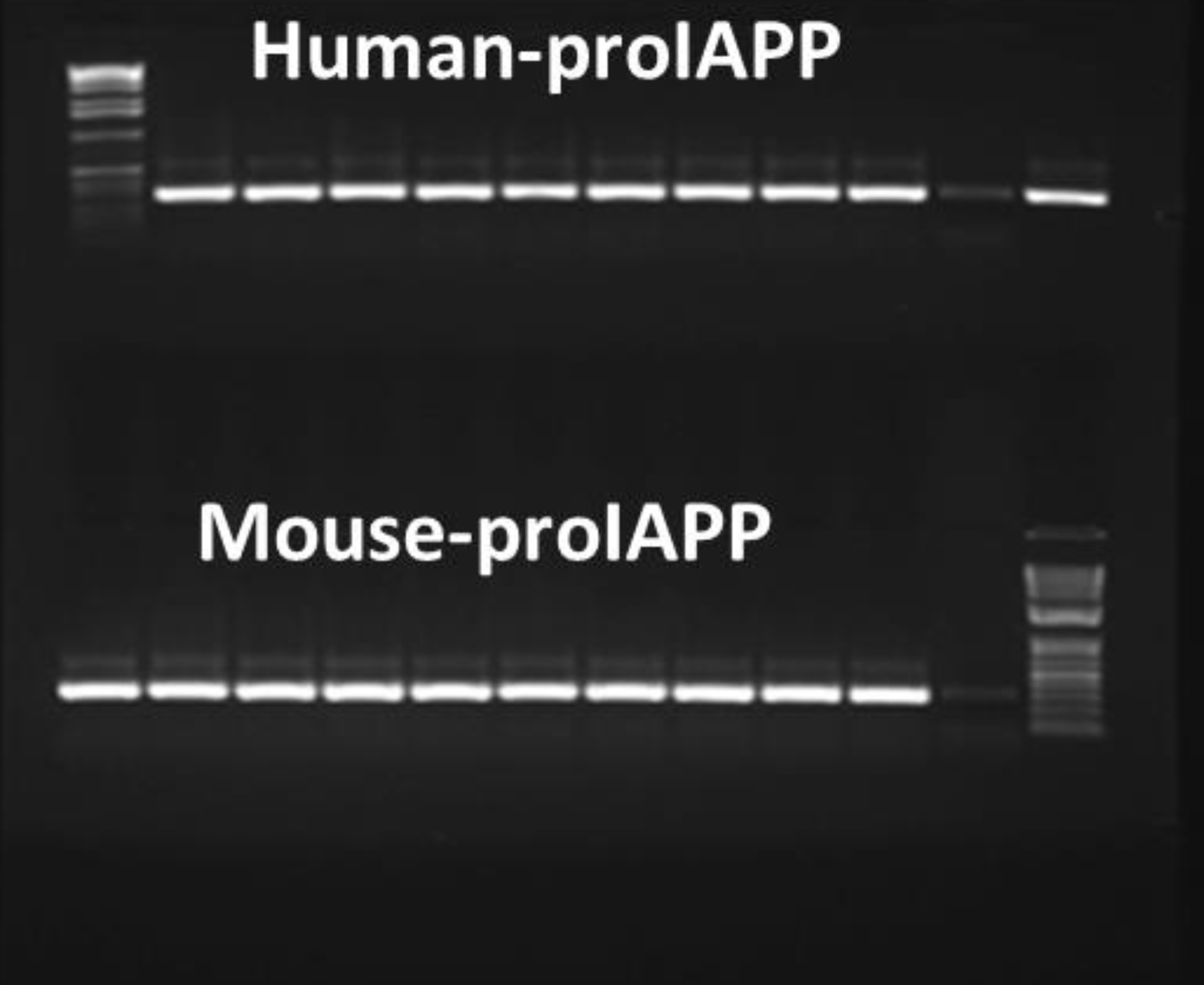

Supplement: S2 Fig — Top panel; lane 1, 1-kb DNA ladder; lane 2–11, DNA extracted from various h-proIAPP colonies; lane 12, h-proIAPP vector (Origene) as a positive control. Bottom panel; lane 1–10, DNA extracted from different m-proIAPP colonies; lane 11, pCMV6 (Origene) as a negative control; lane 12, 1-kb DNA ladder. (TIF) [file pone.0149409.s004.tif]

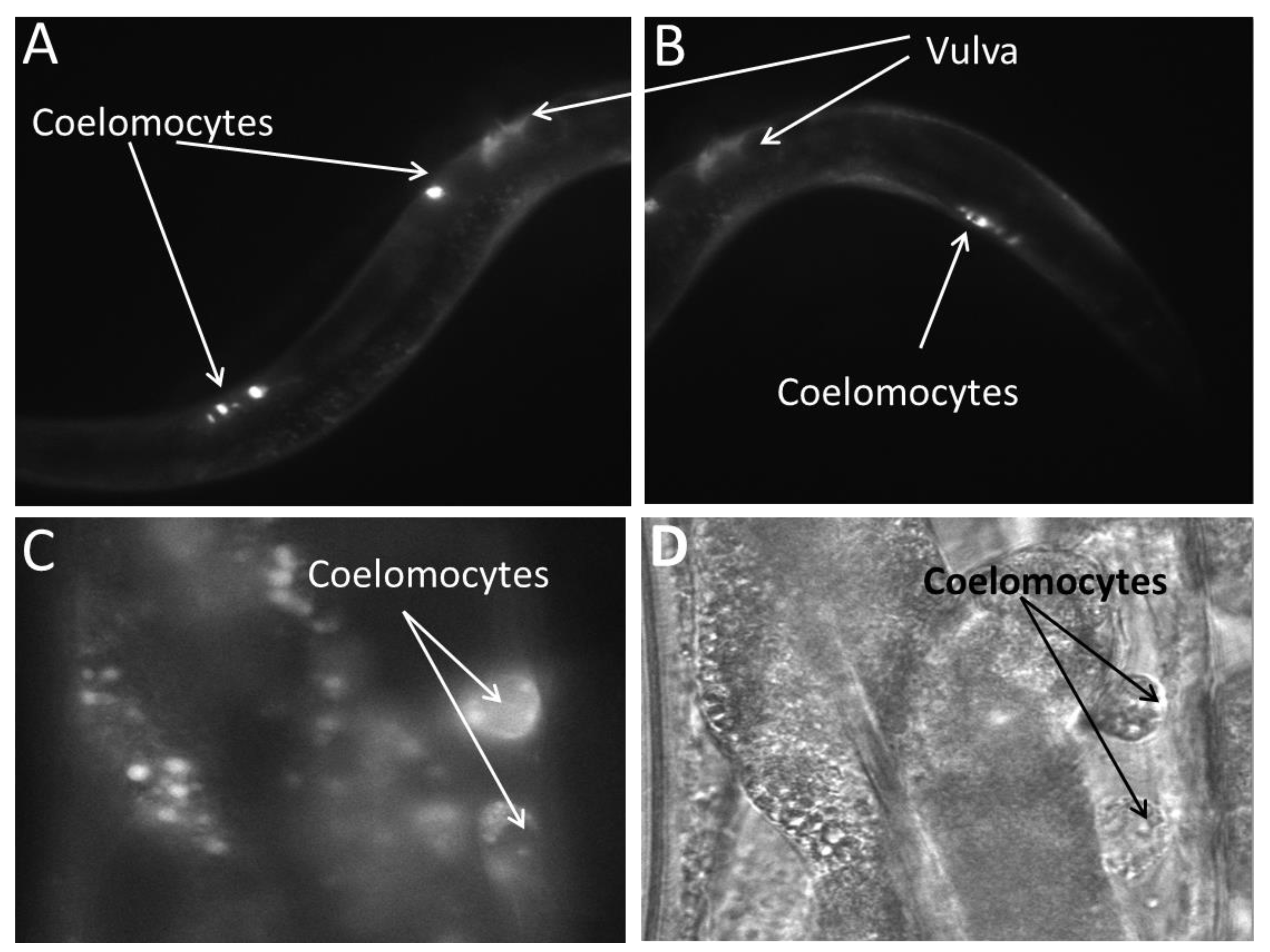

Supplement: S3 Fig — A. h-proIAPP adult C. elegans ventral coelomocytes and B. Dorsal coelomocytes images obtained with a fluorescent microscope at 40X magnification. Coelomocytes are suggested to be phagocytic and similar in function to the macrophages of vertebrates. They have a relatively fixed position in the body cavity. C. h-proIAPP coelomocytes fluorescence images and D. Phase contrast images using 100X magnification. (TIF) [file pone.0149409.s005.tif]

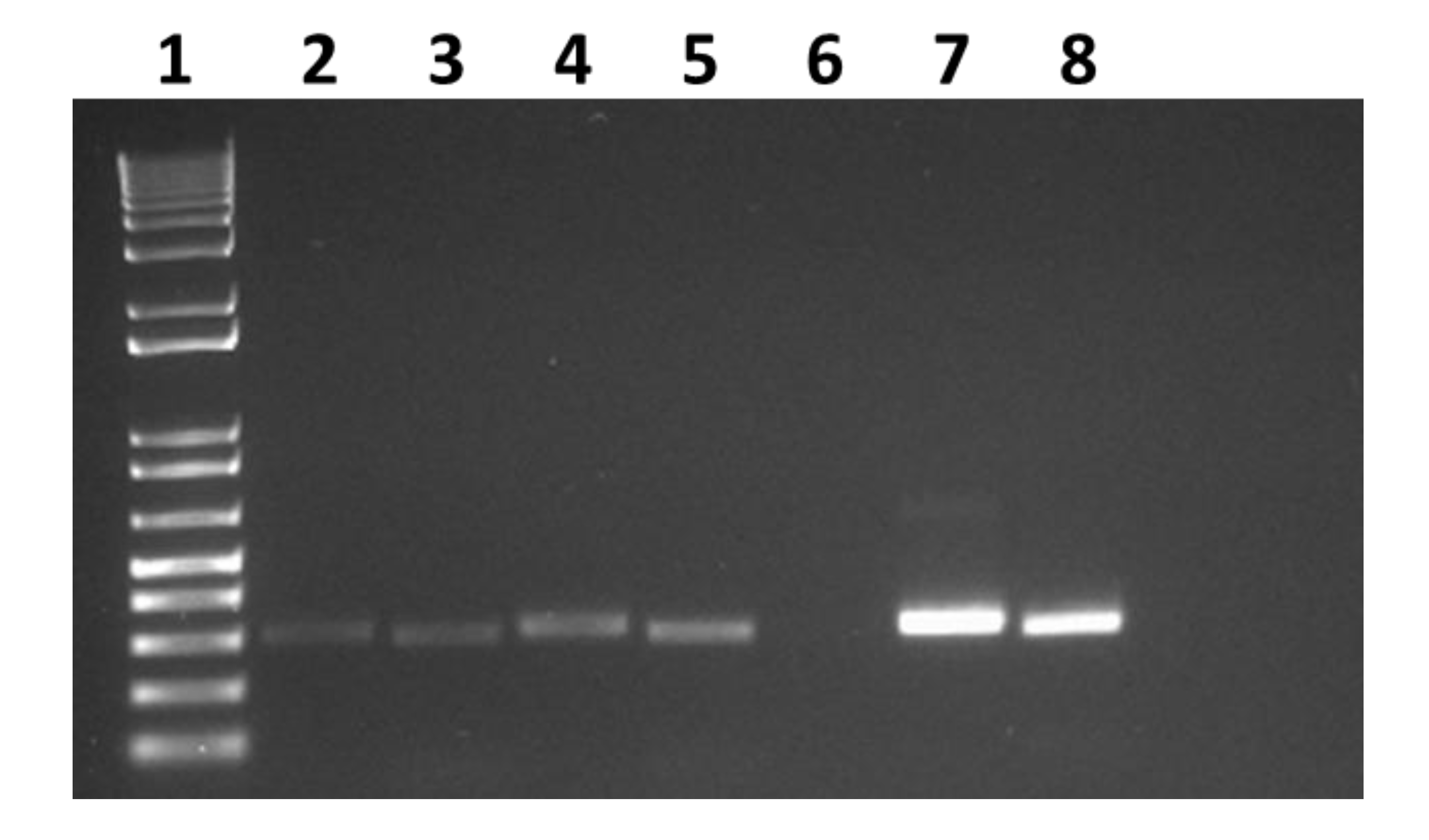

Supplement: S4 Fig — RT-PCR was performed on h-proIAPP and m-proIAPP worms and PCR samples were taken at 25, 27 and 29 cycles of amplification and semi-quantified by visualization following electrophoresis in 1.5% agarose gels. Lane 1, 1-kb DNA ladder; lane 2, PCR product taken at 25 cycles of amplification of m-proIAPP gene; lane 3, PCR product taken at 25 cycles of amplification of h-proIAPP gene; lane 4, PCR product taken at 27 cycles of amplification of m-proIAPP gene; lane 5, PCR product taken at 27 cycles of amplification of h-proIAPP gene; lane 6, empty; lane 7, PCR product taken at 29 cycles of amplification of m-proIAPP gene; lane 8, PCR product taken at 29 cycles of amplification of h-proIAPP gene. (TIF) [file pone.0149409.s006.tif]

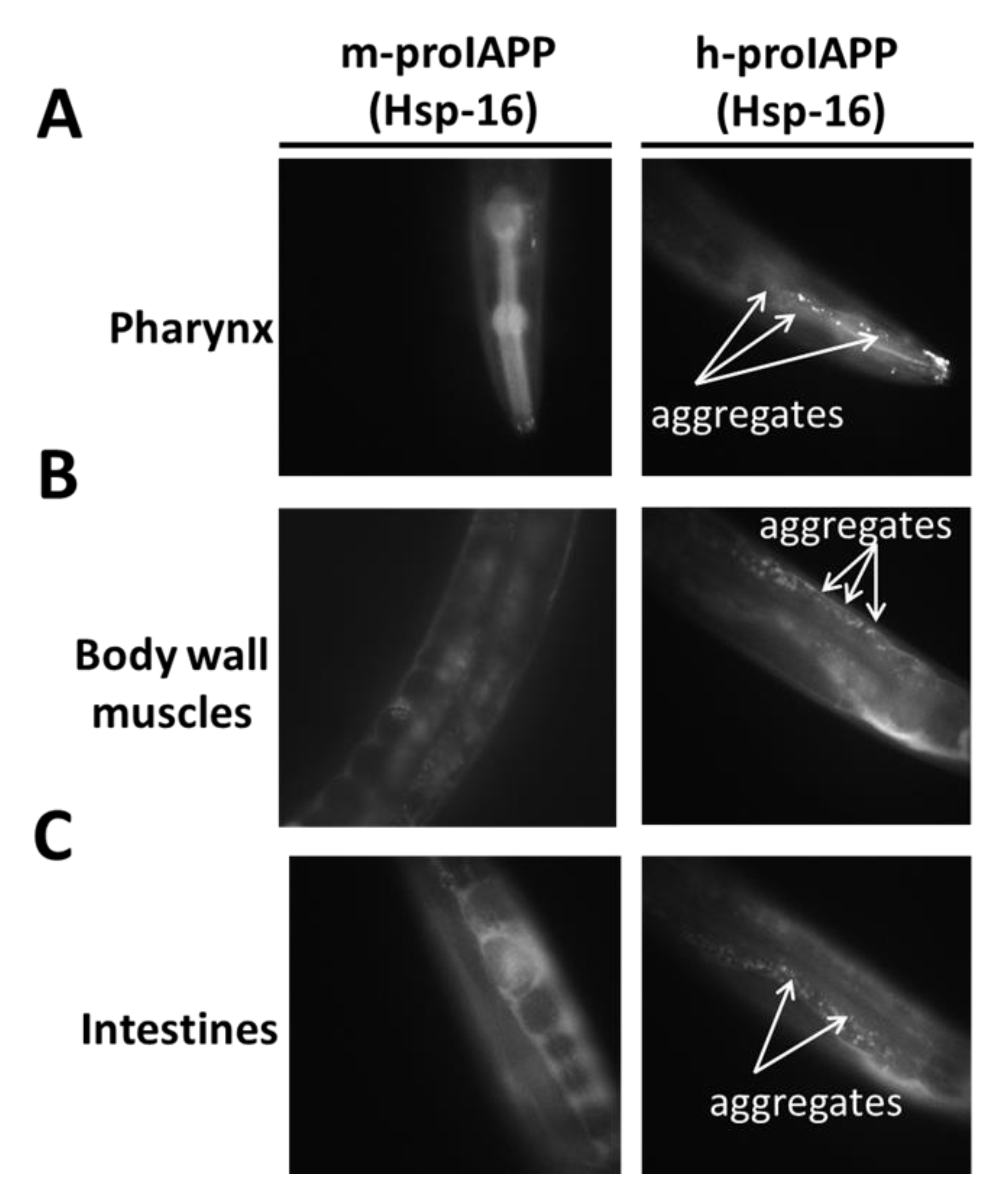

Supplement: S5 Fig — Mouse and human pro-IAPP tagged with YFP expressed under the inducible hsp-16-2 promoter were observed in A, pharynx; B, body wall muscles; C, intestine, after animals were exposed to heat stress at 33°C for 90 minutes. Images were obtained using a fluorescent microscope at 40X magnification. Arrows indicate areas of aggregation. (TIFF) [file pone.0149409.s007.tiff]
